# Small heat shock proteins with two alpha-crystallin domains: a new set of proteins in the earthworm *Eisenia fetida* with differential transcriptional responses to stressors

**DOI:** 10.1007/s11356-026-37811-y

**Published:** 2026-05-11

**Authors:** Natasha Tilikj, Mercedes de la Fuente, Alejandro Martínez Navarro, Jose-Luis Martínez-Guitarte, Marta Novo

**Affiliations:** 1 Biodiversity, Ecology and Evolution Department, Faculty of Biological Sciences, Complutense University of Madrid, C/José Antonio Nováis 12, 28040 Madrid, Spain; 2 Group of Invertebrates Bioinformatics and Molecular Ecotoxicology, Facultad de Ciencias, UNED, Av. Esparta S/N, 28232 Las Rozas, Madrid, Spain

**Keywords:** Earthworms, Environmental stress, Stress biomarkers, Heat shock response, Climate change, Adaptation

## Abstract

Climate change and environmental pollution are two primary challenges facing biodiversity and ecosystem stability. Earthworms are key contributors to soil structure and nutrient cycling, and their molecular stress responses can provide an early indication of soil health impairment. Heat shock proteins are central to the stress response, and small heat shock proteins (sHSPs) are ATP-independent chaperones that limit stress-induced protein aggregation. Because their expression is stress-sensitive, sHSPs are promising molecular markers for soil stress and contributors to thermotolerance. *Eisenia fetida*, a widely used ecotoxicology model, relies on molecular chaperones like small heat shock proteins (sHSPs) for stress tolerance. We previously characterized sHSPs containing a single α-crystallin domain (ACD) in *E. fetida.* Here, we report the first identification of sHSPs containing two α-crystallin domains (ACDs) in annelid species. These genes were identified from an *E. fetida* transcriptome, their domain architecture was defined, and their transcriptional responses were quantified under heat stress, desiccation, and exposure to two pollutants (bisphenol A and endosulfan), including combined exposure with elevated temperature. Double-ACD sHSPs showed stimulus- and time-dependent transcriptional patterns. Moderate heat and desiccation primarily induced late (24 h) upregulation of several sHSP genes, whereas bisphenol A at optimal temperature did not result in significant transcriptional change and endosulfan produced only limited changes under single-stressor exposure. In contrast, combined exposure to endosulfan and elevated temperature triggered a significant upregulation of multiple sHSP genes, consistent with an additive stress effect. These results expand this protein family diversity in annelids and support a staged sHSP response in which structurally distinct sHSPs may contribute to resilience under prolonged or combined environmental stress.

**Supplementary Information:**

The online version contains supplementary material available at 10.1007/s11356-026-37811-y.

## Introduction

In the Sixth Assessment Report (AR6), the Intergovernmental Panel on Climate Change (IPCC) emphasizes that ongoing climate change poses a severe threat to living organisms (Parmesan et al., 2022). Chemical pollution adds an additional layer of stress, including metals, pesticides, plastic additives, and other endocrine-disrupting compounds (Chen et al. 2025; Yu et al. 2019). Despite long evolutionary exposure to natural toxins and environmental variability, current pressures differ in magnitude, mixture complexity, and rate of change (Moe et al. 2013; Rillig et al. 2023). A key question is whether stress-response capacity and physiological plasticity are sufficient under increasingly frequent combined stressors.

To assess how organisms cope with environmental stressors, it is essential to first identify the mechanisms they employ. Among these, the stress response is a central pathway activated when environmental conditions change and disrupt cellular homeostasis. Heat shock proteins (HSPs), first described by Ritossa in the 1960s (Ritossa 1962, 1996), are principal components in this response. These proteins were originally believed to be triggered by heat, leading to their designation as heat shock proteins. However, later research showed that heat shock proteins are a conserved, stress-responsive chaperone network involved in many core cellular processes: they bind nascent and stress-damaged proteins, promote correct folding and refolding, prevent aggregation, assist protein-complex assembly, and help route irreversibly misfolded proteins to degradation pathways, thereby maintaining proteostasis under both stress and normal conditions (Hu et al. 2022; Tutar & Tutar 2010). Their expression can be induced by a wide range of stressors; for example, Jeyachandran et al. (2023) reviewed HSP responses in aquatic organisms under desiccation/osmotic stress as well as exposure to heavy metal and endocrine disruptors, while Banfi et al. (2025) and Swart et al. (2022) reported comparable, multi-stressor HSP responses across terrestrial invertebrates. HSPs are divided into different families depending on the size and the domains they present, primarily including large HSPs, HSP90, HSP70, HSP60, HSP40, and small HSP families (Hu et al. 2022). In this work, we have centered our attention on small HSPs, the family of proteins that shows more diversity between species in number and sequence (Franck et al. 2004).

They are characterized by a highly conserved α-crystallin domain (ACD), the hallmark of the family, and disordered N- and C-terminal regions (Gusev et al. 2002). These proteins act as ATP-independent molecular chaperones that bind partially unfolded proteins, limiting irreversible aggregation and supporting subsequent refolding or clearance (Miller & Reichow 2025). Variation in sHSP domain architecture, including proteins with multiple ACDs, may change interaction surfaces and oligomer dynamics, thereby influencing substrate binding and potentially supporting different roles during early versus prolonged stress (Bagnéris et al. 2009; Santhanagopalan et al. 2018). In recent years, they have garnered attention because of their diversity and rapid evolution compared to the other HSPs, the different roles in cellular processes and disease, and their potential as biomarkers (Carra et al. 2019; Ecroyd et al. 2023; Gupta et al. 2010). Vertebrate sHSPs, primarily in humans, have been studied due to their medical relevance, whereas invertebrate sHSPs remain understudied, despite the extensive number of animal groups and diversity of physiological adaptations they exhibit. While some of them have been described in rotifers (Park & Kwak 2014), insects (Li et al. 2020; Ruan et al. 2022), crustaceans (Zhang et al. 2023), and mollusks (Lei et al. 2016; Sun & Hu 2016), some groups remain poorly studied. Annelida is one of these groups, and although many putative sHSPs have recently been identified in silico (de la Fuente & Novo 2022), experimental evidence on their roles is still lacking.

The earthworm *Eisenia fetida* is a poikilotherm that depends on the environmental conditions. Humidity and temperature are two primary factors that can alter its metabolism (Diehl & Williams 1992). Although it is an epigeic species (lives in superficial soil layers), OECD guidelines recommend *E. fetida* as a standard model for earthworm ecotoxicity testing because it is easy to culture and yields reproducible responses that are considered broadly comparable to soil-inhabiting species (OECD 1984, 2016). Accordingly, it is widely used to quantify sublethal molecular and physiological responses to diverse contaminants, including pesticides, metals, and endocrine-disrupting compounds (Wang et al. 2015; Riedl et al. 2022; Chen et al. 2025). Human activities are increasing chemical contamination, exposing earthworms to pollutants in their natural habitat. At the same time, climate change is altering soil temperature and moisture regimes, making it important to examine how earthworms respond to both single and combined stressors. Combined exposures are ecologically relevant because soil organisms often experience chemical contamination alongside thermal and moisture fluctuations; these co-occurring stressors can reshape energetic allocation and proteostasis demands compared with single stressors (Rillig et al. 2023; Narváez et al. 2022; Schäfer et al. 2023). Understanding these responses is essential for protecting ecosystem functions supported by earthworms, including agricultural productivity, carbon cycling, and the soil resilience to disturbance (Blouin et al. 2013; Fonte et al. 2023). By analyzing the transcriptome of *E. fetida*, several sHSP proteins were identified. de la Fuente and Novo (2022) classified sHSPs based on the α-crystallin domain (ACD) phylogeny and the number of ACDs, defining three clusters of ACDs—identified as A, B, and C. Interestingly, certain sHSPs in *E. fetida* exhibit unique architectures, containing two or more ACDs. Single-ACD sHSPs in *E. fetida* were recently characterized under the same general exposure framework (temperature/moisture variation and chemical contact tests; Tilikj et al. 2025), enabling direct comparison of transcriptional timing between single-ACD and multi-ACD sHSP families in current work. Here we focus on sHSPs with multiple ACDs and analyze their functional response to heat, desiccation, as well as bisphenol A and endosulfan, including combined exposure with elevated temperature to model co-occurring climate and pollution stressors.

The objectives of this work are twofold: First, to perform an in silico characterization of the sequences and structures and explore features that may influence their functionality. Second, to compare their transcriptional responses with previously characterized single-ACD sHSPs across stressors and time, assessing their potential as molecular indicators of chemical exposure under co-occurring soil stress.

## Materials and methods

### Gene identification and characterization: sequence analysis and structural predictions

Nine *Eisenia fetida* transcripts previously predicted by de la Fuente and Novo (2022) were chosen based on the criteria that they encode two or more ACDs. To identify the most similar protein sequences from well-characterized reference species, SmartBLAST (https://blast.ncbi.nlm.nih.gov/smartblast/smartBlast.cgi) and standard BLAST searches in the NCBI databases (https://blast.ncbi.nlm.nih.gov/) were conducted (Altschul et al. 1990; Sayers et al. 2022). The Batch CD-Search tool was used to detect conserved domains in the protein sequences (https://www.ncbi.nlm.nih.gov/Structure/bwrpsb/bwrpsb.cgi) (Marchler-Bauer et al. 2017; Wang et al. 2023).

A range of protein parameters—including molecular weight, theoretical isoelectric point (pI), instability index, aliphatic index, and GRAVY score—were calculated using the ProtParam tool from ExPASy (http://web.expasy.org/protparam/; Gasteiger et al. 2003). The melting temperature of each protein was estimated with DeepStabP (https://csb-deepstabp.bio.rptu.de/) (Jung et al. 2023). Predictions for subcellular localization were obtained using two web-based tools: (1) BUSCA (http://busca.biocomp.unibo.it/), which integrates various predictors including DeepSig, TPpred3, PredGPI, BetAware, ENSEMBLE3.0, BaCelLo, MemLoci, and SChloro (Savojardo et al. 2018) and (2) DeepLoc-2.1 (https://services.healthtech.dtu.dk/services/DeepLoc-2.1/) (Odum et al. 2024).

Each protein’s 3D structure was modeled using AlphaFold 2.1 within ChimeraX version 1.8 (2024-06-10) (Goddard et al. 2018; Meng et al. 2023; Mirdita et al. 2022; Pettersen et al. 2021), with default settings while enabling PDB template use (https://www.rbvi.ucsf.edu/chimerax). A schematic overview of the analytical workflow is provided in Supplementary File S1 (Figure S1).

For phylogenetic reconstruction, 38 ACD sequences from five taxa were analyzed (*E. fetida* *n* = 26; *Homo sapiens* *n* = 5; *Caenorhabditis elegans* *n* = 3; *Drosophila melanogaster* *n* = 3; *Arabidopsis thaliana* *n* = 1); the *E. fetida* set comprised 21 ACDs from multi-ACD sHSPs (nine dimeric proteins plus EfsHSP68 with three ACDs) and five monomeric sHSP ACDs. In our analysis, each ACD from multi-ACD sHSPs was extracted and treated as an independent phylogenetic unit, and ACD placement was interpreted using the A, B, and C cluster framework defined by de la Fuente and Novo (2022). These sequences were aligned, analyzed and then manually adjusted using ClustalW within the MEGA 11.0.13 program (Tamura et al. 2021), using default settings. The adjustment was performed considering the predicted structural information. The optimal amino acid substitution model was determined using Modeltest-NG (Darriba et al. 2020; Flouri et al. 2015), identifying LG + I + G4 as the best-fit model. Maximum likelihood (ML) phylogenetic analyses of protein sequences were performed with RAxML-HPC BlackBox 8.2.10 (Stamatakis 2014), implemented via the CIPRES Science Gateway (Miller et al. 2010). The best-scoring tree was inferred under the selected model, and support values were estimated using 1000 replicates of the rapid bootstrapping algorithm. The phylogenetic tree was visualized and edited using iTOL v.6.1.1 (Letunic & Bork 2007, 2021).

### Animals

The earthworms used in the experiments belong to a unique genetic lineage of *E. fetida* maintained long-term as a laboratory culture by Dr. Domínguez from the University of Vigo. Stock identity was verified by genotyping (e.g., COI barcoding/COI plus 28S; methods consistent with Pérez-Losada et al. 2005) and maintained by the Zoology Group at the Complutense University of Madrid. Culture conditions were 80% soil moisture, dark and at 21 ± 0.5 °C. Horse manure used for microcosm preparation was chemically untreated and was defaunated by freezing at − 20 °C for 14 days. Only adults (clitellated) were selected for the experiments. Prior to exposure, they were washed with distilled water, dried on filter paper and weighed.

### Heat and desiccation stress treatments

Two physical stressors, heat stress and desiccation stress, were selected based on their relevance to predicted climate change impacts on earthworms, as outlined in the review by Singh et al. (2019), which highlights temperature and soil moisture as key factors affecting earthworm physiology and activity. To prevent lethal bacterial exposure, glass jars containing 5 g of untreated manure were incubated at 21 °C for 24 h before introducing the earthworms. The experimental design used in this study mirrors the one from Tilikj et al. (2025). For heat stress experiments, the earthworms were placed in the prepared jars and exposed to 31 ± 0.5 °C in culture chambers for up to 24 h as a moderate heat stress. In a separate experiment, they were subjected to 35 ± 0.5 °C for 2 h to simulate sublethal stress. Both exposures were at 80% soil moisture (as defined in the microcosm setup). Moisture was verified by mass (weighing), and no measurable mass loss occurred during the 24 h exposure.

Desiccation stress, on the other hand, was induced by exposing the earthworms to two different soil moisture levels, 10 and 20%, at 21 ± 0.5 °C. The exposure time was up to 24 h, with sample collection at 2, 7, and 24 h for both heat and desiccation treatments. Ten animals were exposed per time and condition, while the control was maintained at 21 ± 0.5 °C in culture chambers and 80% soil moisture for the same duration. Survival for heat and desiccation treatments was assessed at each sampling time by checking responsiveness, and no mortality occurred during the exposure period. Moisture was verified by weighing, with no measurable mass loss over 24 h. All individuals were snap-frozen at −80 °C. Eight individuals per condition were used as true biological replicates for RNA extraction and qPCR, while the remaining were reserved as backup.

### Chemical stress by contact test

Chemical stress was induced by using two well-known toxicants: the organochlorine insecticide Endosulfan (END, CAS 115-29-7, Fluka Analytical) and the plasticizer Bisphenol A (BPA, CAS 80-05-7, purity > 99% Sigma Aldrich). Endosulfan (250 g/L in acetone) and Bisphenol A (2 g/L in ethanol) stock solutions were diluted to achieve the desired final concentrations. The contact exposure followed the method described by Novo et al. (2018). Two filter papers soaked with 1 mL of 0.2 g/L BPA or 0.1 g/L END were placed on a Petri dish, until complete evaporation (ethanol or acetone). Later, 2 mL of dH_2_O and one earthworm per Petri dish were added. Solvent controls (filter papers treated with acetone or ethanol, evaporated as in exposure plates) were run in parallel and did not differ from water controls; therefore, water controls are presented. The exposure time was 2 and 7 h at 21 ± 0.5 °C or 26 ± 0.5 °C in culture chambers. Concentrations (0.2 g/L BPA; 0.1 g/L END) were selected based on preliminary sublethal assays (Tilikj et al. 2024) that produced measurable transcriptional responses without acute mortality. These doses were chosen to represent high-end contact exposures, consistent with reports of BPA in landfill leachates (Yamamoto et al. 2001) and endosulfan in contaminated soils (Fang et al. 2016). Eight previously acclimated earthworms from the source culture were used per treatment. Survival was assessed at each sampling time by checking responsiveness, and no mortality occurred during the exposure period. All individuals were snap-frozen at −80 °C. Six individuals per time and condition were used as true biological replicates for RNA extraction and qPCR, while the remaining were reserved as backup. The samples were maintained at −80 °C until processing.

### RNA extraction and retrotranscription

For RNA extraction, the whole earthworm was homogenized to a fine powder using a mortar and a pestle on dry ice. Subsequently, the powder was added to the TRIzol reagent (Invitrogen), and the RNA was extracted following the manufacturer’s instructions. An RNase-free DNase I (Roche) treatment was carried out to prevent DNA contamination. The enzyme was removed by a phenol–chloroform-isoamyl alcohol extraction using Phase Lock Gel Light tubes (5PRIME). RNA integrity was assessed by gel electrophoresis and only samples with intact RNA bands were used. Quantification was done with a NanoDrop One Spectrophotometer (Thermo Scientific), and the samples were diluted to the adequate concentration for retrotranscription. For retrotranscription, 1 μg of total RNA was incubated with 200 units of the M-MLV enzyme (Invitrogen), 0.5 μg oligo dT20 primer (Biotools), 0.5 μg random hexamers (Biotools), and 10 mM dNTPs (Biotools) in a final reaction volume of 40 µl. After 50 min at 37 °C, the reaction was stopped by heating at 70 °C for 15 min. The cDNA samples were stored at −20 °C until use.

### Real-time polymerase chain reaction (PCR)

Gene expression was analyzed by Real-Time PCR. The primers were designed with the identified sequences using the Primer-Blast tool (https://www.ncbi.nlm.nih.gov/tools/primer-blast/index.cgi) for amplification at the same temperature. The primer data are shown in Table S1 of the Supplementary File S2. The specificity of amplification and the efficiency calculations were done in the same way as in Tilikj et al. (2024). Briefly, efficiency was obtained by five serial 1:2 dilutions from a cDNA mix of the previously obtained cDNAs. Real-Time PCR was performed in a final volume of 10 µl. The master mix was prepared with 10X buffer (Biotools) and 1 µl of 10 mg/mL bovine serum albumin (BSA, Sigma). The primers were used at a final concentration of 550 nM each, and 500 ng of cDNA was amplified. The Taq polymerase was from Biotools. The PCR program was 95 °C for 5 s and 35 cycles of 95 °C for 5 s, 62 °C for 10 s, and 65 °C for 15 s. A melting curve analysis (65 °C for 5 s and 95 °C for 5 s) was performed to ensure the specificity of the amplicons.

As reference genes, Actin-β (*Act*), ribosomal protein L11 (*rpL11*), and the TATA-binding protein (*TBP*) genes were used (primers are described in Tilikj et al. 2024). The gene expression was determined by the 2^−ΔΔCt^ (Pfaffl, 2001).

### Statistical analysis

Data were assessed for normality using the Shapiro–Wilk test and were non-normally distributed. Consequently, the differences between the treatments and the control were analyzed by a nonparametric Kruskal–Wallis test, performed with the SPSS 24 software (IBM, USA). Kruskal–Wallis tests were followed by pairwise post hoc comparisons using Dunn’s test with Bonferroni correction for multiple testing. In all cases, *p*-value < 0.05 was considered significant.

## Results and discussion

### Double-ACD sHSPs in *E. fetida*: sequence and structural analysis

To determine the potential functions of the proteins encoded by the identified genes, we performed the following analyses: (1) identification of homologous sequences; (2) detection of conserved domains within the protein sequences; (3) calculation of physicochemical properties and (4) prediction and visualization of AlphaFold 3D structures (Fig. S1). These analyses provide initial evidence for functional classification, particularly as small heat shock proteins. The results are summarized in Tables 1 and 2, while 3D structures can be found in the Supplementary file Dataset S1. This dataset includes the most reliable AlphaFold model (ranked_1) for each protein, displayed using per-residue model confidence scores (pLDDT), together with Predicted Aligned Error (PAE) plots for all five AlphaFold-generated models and an additional structurally plausible model selected based on PAE patterns. Functional predictions based on these analyses were subsequently supported by qPCR experiments assessing gene expression under stress conditions.

**Table 1:** Gene and basic genetic information

Gene	Former identifier ^1^	Sequence completeness	GeneBank accession number	Top BLAST match ^2^	Transcript length (nt) ^3^	Protein length (aa) ^4^
EfsHSP32.doubleACD	Ef_ID1	Complete	PQ603076	GIKG01088656	855	284
EfsHSP38.1.doubleACD	Ef_ID3	Complete	PQ603077	GIKG01098320	1020	339
EfsHSP38.7.doubleACD	Ef_ID5	Incomplete	PQ603078	GIKG01013943	1023*	340*
EfsHSP55.doubleACD	Ef_ID6	5' Incomplete	PQ603079	GIKG01130900	1464*	487*
EfsHSP33.doubleACD	Ef_ID10	Complete	PQ603080	GIUK01088083	876	291
EfsHSP59.doubleACD	Ef_ID11	5' Incomplete	PQ603081	GIKG01136737**	1578†	525†
EfsHSP84.doubleACD	Ef_ID12	5' Incomplete	PQ603082	GIUK01042923	2247*	748*
EfsHSP52.doubleACD	Ef_ID13	Complete	PQ603083	GIKG01145199	1407	468
EfsHSP36.doubleACD	Ef_ID15	Complete	PQ603084	GIKG01094581	1023	340

^1^Data from de la Fuente and Novo (2022).

^2^Incomplete sequences from the BLAST search are marked with a double asterisk (**).

^3^^,4^Transcript length (nt) and protein length (aa) values marked with an asterisk (*) are estimated based on the top BLAST match for incomplete sequences, except for EfsHSP59.doubleACD, which is marked with a dagger (†). In this case, the transcript length was estimated from our own sequence due to the incompleteness of the top BLAST match.

**Table 2:** Physicochemical properties of dimeric sHSPs

Protein name	MW ^5^	pI ^6^	Protein GRAVY ^7^	Instability index ^8^	Aliphatic index ^9^	Subcellular localization ^10^	Tm (21 °C)/Tm (31 °C) ^11^	
EfsHSP32.doubleACD	32.23	4.91	−0.657	48.14	86.8	Cytoplasm/nucleus	42.56 °C/42.71 °C	
EfsHSP38.1.doubleACD	38.1	6.12	−0.826	55.75	64.37	Cytoplasm/nucleus	42.92 °C/43.01 °C	
EfsHSP38.7.doubleACD	38.7*	6.43*	−0.795*	51.950*	62.3*	Cytoplasm/nucleus*	41.95 °C/42.10 °C*	
EfsHSP55.doubleACD	55.5*	5.37*	−0.909*	50.15*	57.84*	Cytoplasm/nucleus*	52.87 °C/53.52 °C *	
EfsHSP33.doubleACD	33.21	4.90	−0.608	45.47	76.94	Cytoplasm/nucleus	38.32 °C/38.35 °C	
EfsHSP59.doubleACD	58.97†	8,23†	−0.691†	49.28†	67.73†	Nucleus†	44.95 °C/46.45 °C†	
EfsHSP84.doubleACD	83.94*	9.36*	−1.071*	55.87*	52.01*	Cytoplasm/nucleus*	41.07 °C/41.55 °C*	
EfsHSP52.doubleACD	52.24	8.79	−0.842	38.44	62.65	Cytoplasm/nucleus	40.68 °C/41.91 °C	
EfsHSP36.doubleACD	36.76	5.1	−0.544	40.69	75.74	Cytoplasm/nucleus	42.56 °C/42.71 °C	

For incomplete sequences, molecular properties (^5^,^6^,^7^,^8^,^9^,^10^,^11^) are determined based on the top BLAST match and indicated with an asterisk (*). An exception is EfsHSP59.doubleACD, marked with a dagger (†), where molecular properties were determined from the EfsHSP59.doubleACD sequence itself due to the incompleteness of the top BLAST match.

BLAST finds similar sequences to infer possible functions. A standard nucleotide BLAST search of the nine EfsHSP transcripts, purported to contain a double-ACD region, was conducted using the Transcriptome Shotgun Assembly database (accessed on 10/10/2024). The analysis revealed that four of the previously identified transcripts were incomplete. The results of the BLAST search are summarized in Table 1, with the top BLAST hits determined based on query coverage and percentage identity. CD-search revealed that seven of the sequences contained two conserved regions with at least one of them being a metazoan ACD region (Supplementary File S2). However, two notable exceptions were observed: EfsHSP83.doubleACD, for which the CD-search analysis revealed only a single identifiable ACD region, likely due to lower sequence conservation of the second ACD region, and EfsHSP59.doubleACD, which exhibited three conserved regions, including the DNA-binding THAP domain.

Physicochemical analysis of the sHSPs with two ACD domains are summarized in Table 2. Following the nomenclature in Tilikj et al. (2025), molecular weight (MW) was used to assign new identifiers for the proteins used in this study. Theoretical isoelectric points (pI) may be indicative of subcellular localization, potential interactions with other molecules, and, ultimately, the functional roles of the proteins (Tokmakov et al. 2021; Salis et al. 2011). In the case of the sHSPs with multiple ACD domains analyzed in this study, all appear to be cytoplasmic or nuclear proteins, with EfsHSP59.doubleACD standing out as a protein for which the predictions clearly suggest a nuclear localization. Monomeric sHSPs share subcellular localization with dimeric sHSPs and are primarily found in the cytoplasm and nucleus (Tilikj et al. 2025). Cytoplasmic and nuclear sHSPs have also been described in the cardiac tissues of rats and humans, as well as in muscle tissues of humans (Mymrikov et al. 2011; Pipkin et al. 2003). Most of the proteins exhibit a slightly acidic pI, ranging from 4.9 to 6.4, while three, EfsHSP84.doubleACD, EfsHSP59.doubleACD, and EfsHSP52.doubleACD, are basic, which may affect their interactions or stability in different cellular environments (Tokmakov et al. 2021). Negative GRAVY scores reflect the hydrophilic nature of the sHSPs with two ACD domains, which indicates they are soluble proteins (Lanneau et al. 2008). In relation to the predicted melting temperatures (Tm), most of the proteins exhibit Tm values ranging between 40 and 45 °C. Higher Tm can be indicative of functional stability under elevated temperatures, although it is not the sole determinant of gene expression or stress responsiveness (Gehring and Wehner 1995; Ku et al. 2009). Notably, EfsHSP55.doubleACD stands out with a higher Tm, ranging from 52.8 to 53.5 °C, while EfsHSP33.doubleACD shows the lowest Tm at 38 °C. This lower Tm for EfsHSP33.doubleACD is inconsistent with a supposed role as a thermal stress protein, as it suggests a reduced stability under heat stress conditions.

To better understand the evolutionary origin and potential functional conservation of the double ACD sHSPs, we investigated sequence similarity and phylogenetic relationships with homologous proteins within metazoa. The search of highly similar proteins with SmartBLAST uncovers homology to the equivalent proteins in *Caenorhabditis elegans*, *Mus musculus*, and *Homo sapiens;* however, due to the protein length (aa) of double-ACD sSHPs, values like query cover and identity are all below 50% (Supplementary File S2).

**Fig. 1:**
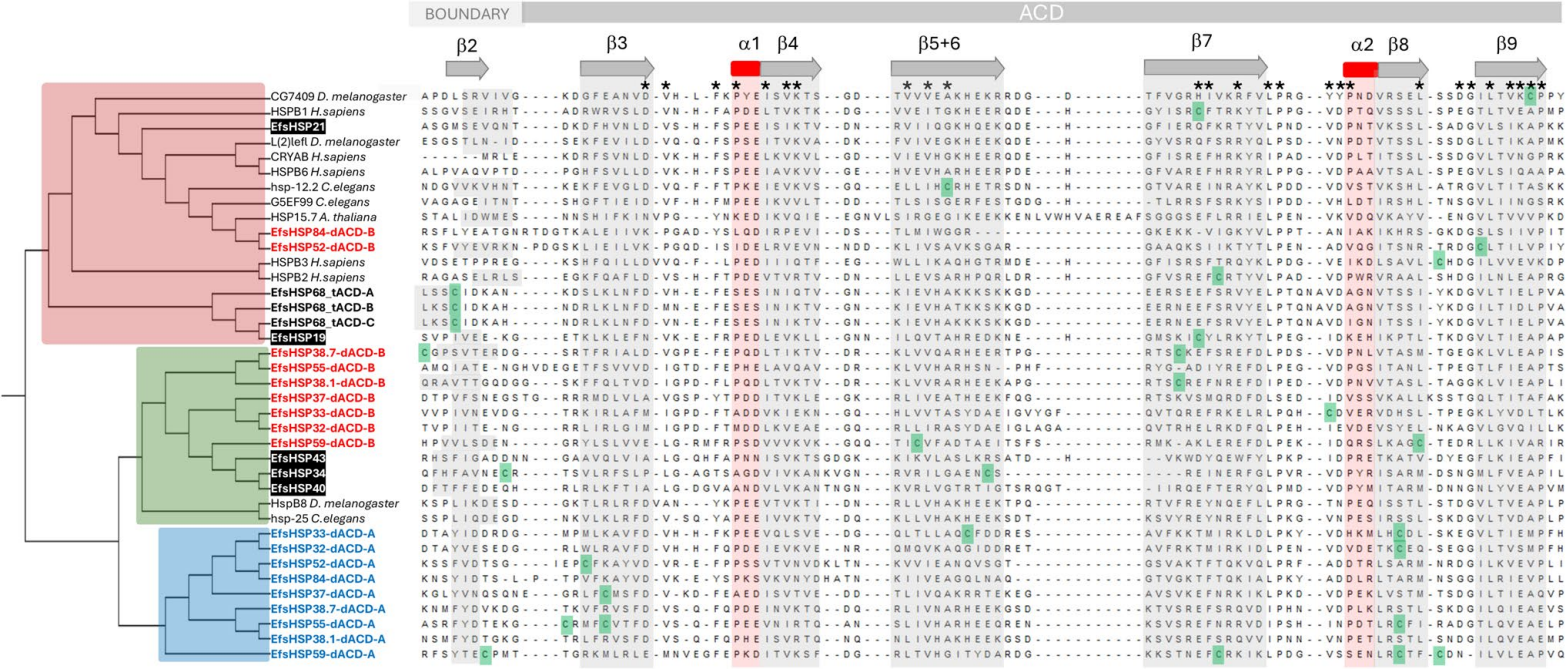
Sequence alignment of the alpha-crystallin domain (ACD) regions from small heat shock proteins (sHSPs) involved in the study. The clustering of the first ACD regions (blue letters) suggests a shared evolutionary origin. The second ACD regions (red letters) in sHSPs with a dual architecture group with monomeric sHSPs from *E. fetida* (shown with a black background) and the triple ACD sHSPs (shown in bold letters), alongside other well-characterized metazoan sHSPs

Phylogenetic analysis was performed, including the proteins analyzed in this study alongside these homologous proteins found in the SmartBLAST analysis, as well as the previously identified and studied monomeric sHSPs with a single ACD from *E. fetida* (de la Fuente and Novo 2022; Tilikj et al. 2025). The results of this analysis allow the placement of the proteins under study within a broader sHSP phylogenetic context., as shown in Figs. 1 and S2 in DatasetS1. It is important to note that all the first ACD domains of the double-ACD proteins cluster together, which likely reflects a combination of sequence conservation and shared structural constraints, rather than unequivocally indicating a common origin for all these domains prior to the differentiation of the various proteins. The only exception within the proteins with multiple ACDs studied is sHSP68, initially considered a protein with a single ACD (de la Fuente and Novo 2022), but Tilikj et al. (2025) showed that the available transcript was truncated (ORFfinder did not detect a stop codon), and subsequent analysis uncovered the presence of three ACDs. These three ACDs group together, which may be consistent with a more recent triplication event, although the exact duplication history cannot be resolved from ACD-only phylogeny.

AlphaFold models were evaluated using pLDDT and PAE values. Confidence was consistently higher across the ACD cores than in terminal/linker regions, and PAE indicated that inter-domain orientation is less certain than within-domain geometry. The AlphaFold-predicted models for double-ACD sHSPs contain two ACD domains that are structurally comparable to those previously described for monomeric sHSPs (Tilikj et al. 2025; see Supplementary File S2). In our models, the ACD regions display the expected β-sandwich/β-strand-rich fold characteristic of sHSP ACDs, consistent with the sequence and secondary-structure conservation shown in Fig. 1. All predicted structures and associated confidence/PAE outputs for the double-ACD proteins analyzed here are provided in Dataset S1.

Oligomerization is a defining characteristic of sHSPs. Here, AlphaFold predictions were generated for single polypeptide chains containing two ACDs within the same chain, and we did not perform multimer predictions of homo- or hetero-oligomers. Nevertheless, the ACD cores retain the conserved β7 strand that is known to mediate sHSP dimer interfaces through β7–β7 interactions in characterized systems (Haslbeck et al. 2019; Janowska et al. 2019). A notable feature of the double-ACD proteins analyzed here, except EfsHSP59.doubleACD, is an N-terminal α-helix of ~ 35 amino acids, which may act as a flexible interdomain connector between the two ACDs, analogous to the flexible N-terminal region described in plant sHSPs (Lambert et al. 2011; Van Montfort et al. 2001). In EfsHSP59.doubleACD, this region is replaced by a THAP domain (Dataset S1). Finally, EfsHSP59.doubleACD is the only double-ACD sHSP analyzed that contains cysteine residues in the β7 strand (Figure S2; Dataset S1). In human sHSPs, particularly the oxidized form of HSPB1, such cysteines can form disulfide bonds across the dimer interface and modulate chaperone activity under oxidative stress (Rajagopal et al. 2015).

### Changes in double-ACD sHSP gene expression in response to stress

Because domain architecture can influence predicted structural stability and interaction surfaces, we next asked whether double-ACD sHSPs display transcriptional responses that differ in timing and stress specificity from previously characterized single-ACD sHSPs in *E. fetida* (Tilikj et al. 2025). We therefore quantified expression dynamics of nine double-ACD sHSP genes under heat, desiccation, and chemical exposure (bisphenol A and endosulfan), including combined exposure with elevated temperature, across multiple time points.

#### Response to heat stress

**Fig. 2:**
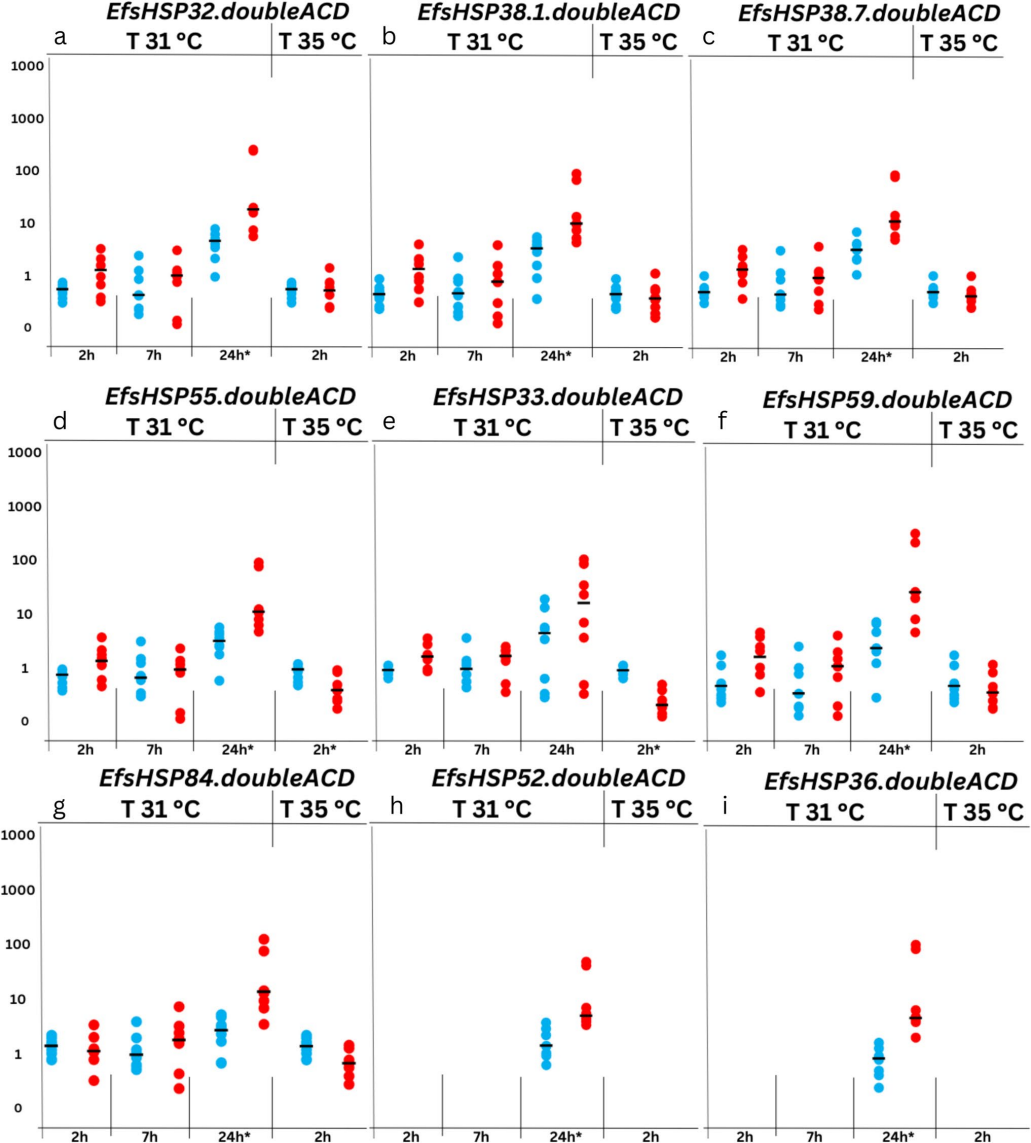
Expression profiles of *EfsHSP* genes with dual ACD domain architecture under heat stress treatments of 31 °C and 35 °C across time points (2 h, 7 h, and 24 h). Missing data in **h** and **i** for the control samples at 2 h and 7 h are due to the RT-PCR amplification not reaching the threshold for detection, resulting in undetermined Ct values (NaN). This reflects the absence of detectable expression under these specific conditions. Asterisks represent significant differences (*p* < 0.05) between control and exposure. Numbers on *y* axis are arbitrary

To investigate the response of the identified small heat shock proteins (sHSPs), their transcriptional activity was analyzed at two temperatures: 31 °C and 35 °C. The results are summarized in Table S2 in Supplementary File S2. At 31 °C, three time points were examined: 2, 7, and 24 h. No significant changes in gene expression were observed at 2 and 7 h (Fig. 2). These findings contrast with the expression patterns observed in monomeric sHSPs in *E. fetida*, where specific monomeric sHSPs, such as EfsHSP19 and EfsHSP21, displayed significant and consistent upregulation at earlier time points (Tilikj et al. 2024). At 24 h, eight genes for dimeric sHSPs demonstrated increased transcriptional activity (Fig. 2), indicating a delayed response to heat stress at this temperature. It is particularly striking that even double-ACD sHSPs which belong in this same phylogenetic cluster as these two monomers have no significant activity at earlier time points. This is further supported by the observation that continuous 24-h heat exposure elicited a two-phase transcriptional response of small heat shock proteins (sHSPs) in *E. fetida*. This temporal separation suggests a potential sequential or synergistic role in thermotolerance: monomeric sHSPs may act as early responders, rapidly engaging in protective functions during the acute phase of heat stress, while double-ACD sHSPs contribute to sustained protection as stress persists. Although based solely on transcriptional profiles, this pattern may reflect a coordinated strategy in which different sHSP subtypes are mobilized at distinct stages of the cellular stress response to enhance resilience to prolonged thermal exposure. Synergistic models of thermotolerance via synergy between HSPs have been suggested in mouse models (Huang et al. 2007).

Earlier transcriptional activity of triple-ACD-containing and septuple-ACD-containing genes has been reported in the whiteleg shrimp, with expression observed as early as 2 h and peaking at 12 h (Zhang et al. 2023). Tilikj et al. (2025) also reported on sHSPs with three ACDs (EfsHSP68) which was found to be upregulated only at 2 h of moderate heat stress.

At the lethal temperature of 35 °C, no induction was observed; moreover, two sHSPs containing two ACD domains, EfsHSP55.doubleACD and EfsHSP33.doubleACD, were downregulated (Fig. 2). Interestingly, these proteins had the highest and lowest Tm, respectively. Since Tm reflects protein stability rather than regulation, we interpret this cautiously, but the downregulation of EfsHSP33.doubleACD at 35 °C could be consistent with a compensatory response when conditions exceed its stability (Parsell and Sauer 1989; Kwon et al. 1996; Lee and Goldberg 2022). Once again, clear differences emerge, as previous studies reported upregulation of two monomeric sHSPs, EfsHSP19 and EfsHSP21, at 2 h under the same temperature. In the case of the whiteleg shrimp, Zhang et al. (2023) demonstrated that triple-ACD and septuple-ACD-containing genes were involved in heat resistance above 36 °C. The results suggest that the sHSPs with two ACDs have a later role in the response to mild temperature stress, probably related to the long-term response more than the acute response.

#### Response to desiccation

**Fig. 3:**
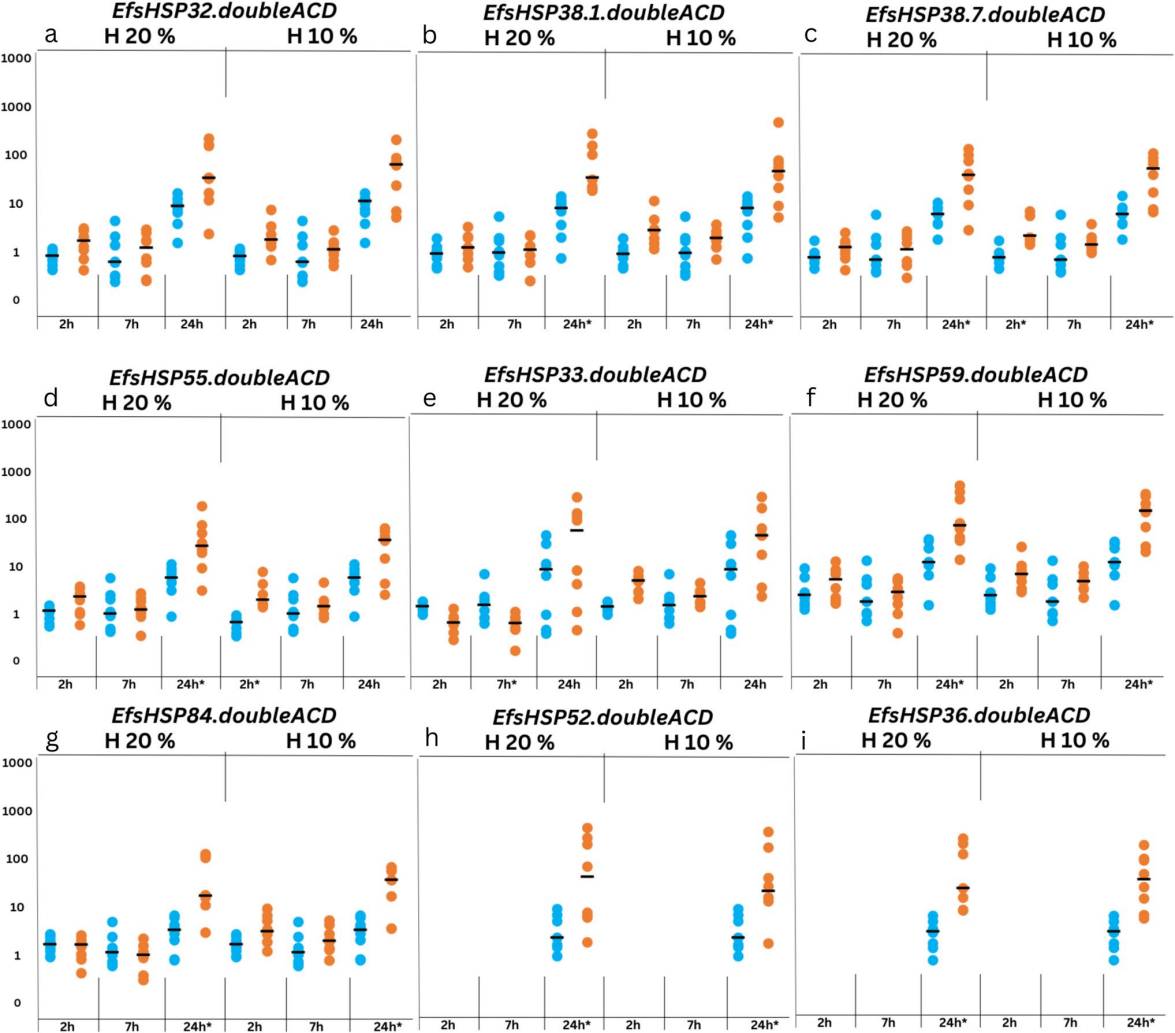
Expression profiles of *EfsHSP* genes with dual-ACD-domain architecture under desiccation stress of H20% and H10% across time points (2 h, 7 h, and 24 h). Missing data in **h** and **i** for the control samples at 2 h and 7 h are due to the RT-PCR amplification not reaching the threshold for detection, resulting in undetermined Ct values (NaN). This reflects the absence of detectable expression under these specific conditions. Asterisks represent significant differences (*p* < 0.05) between control and exposure. Numbers on *y* axis are arbitrary

Desiccation poses a significant challenge for earthworms, as they rely on appropriate soil moisture levels for survival (Singh et al. 2019 and references therein). Prolonged periods of desiccation can suppress earthworm activity and consequently slow down litter decomposition, which affects carbon processing (Rillig et al. 2023; Deng et al. 2021; Martins da Silva et al. 2020). To evaluate earthworms’ response to desiccation, two different soil moisture levels of 10 and 20% were employed. The exposure durations were set at 2, 7, and 24 h, consistent with previous experiments. A summary of the results is shown in Table S2 in Supplementary File S2. The response of double-ACD sHSPs to desiccation stress differs markedly from that of single-ACD sHSPs in *E. fetida*, reflecting distinct roles in managing abiotic stress. Under 20% soil moisture, monomeric sHSPs showed some upregulation at earlier time points (Tilikj et al. 2025). In contrast, sHSPs with two ACD domains exhibited delayed transcriptional modulation, with significant upregulation observed only at 24 h (Fig. 3). An exception was EfsHSP33.doubleACD, which was downregulated at 7 h. At 10% soil moisture, a similar delayed upregulation was observed, with two double-ACD sHSPs exhibiting a staggered transcriptional pattern. EfsHSP38.7.doubleACD and EfsHSP55.doubleACD showed early induction at 2 h, followed by a reduction in activity at 7 h; only EfsHSP38.7.doubleACD showed clear re-induction at 24 h (Fig. 3). This could reflect an early osmotic-shock phase as reported in tardigrades (Hibshman et al., 2023), followed by later dehydration acclimation, but the pattern is clearly supported for only one double-ACD gene. For EfsHSP68, which contains three ACD domains, upregulation was observed only at 2 h and 24 h under 10% desiccation stress, suggesting that its transcriptional response is influenced by the severity of the stress rather than the duration (Tilikj et al. 2025). Overall, the transcriptional patterns of sHSPs with two and three ACD regions show little similarity, indicating they may play fundamentally different roles in the environmental stress response.

In our experiments, double-ACD sHSPs show a similar pattern under heat and desiccation, with induction mainly apparent at later time points. Similar overlap between sHSP responses to heat and desiccation has been reported in anhydrobiotic organisms such as the midge *Polypedilum vanderplanki* (sHSP transcripts are induced during desiccation and are also heat-inducible; Gusev et al. 2011) and in tardigrades, where sHSPs are linked to both desiccation and heat tolerance (Hibshman et al., 2023).

#### Response to chemical stress

The response of double-ACD sHSPs to two well-known contaminants, the plasticizer bisphenol A and the insecticide endosulfan, was analyzed to assess their reaction to chemical stressors (Table S2 in Supplementary File S2). These exposures were conducted at the standard growth temperature (21 °C) and an elevated temperature (26 °C), simulating the worst-case climate change scenario as outlined in the 2022 IPCC report. This report predicts temperature increases ranging from 1.5 °C by 2050 (scenario 1, most optimistic) to 4.4 °C by 2100 (scenario 5, most severe) (IPCC 2022).

In *E. fetida*, sHSPs with single or double-ACDs exhibit distinct responses to individual chemical stressors, yet under the combined stress of BPA exposure and elevated temperatures, both types show limited transcriptional modulation. Among the monomeric sHSPs, only EfsHSP21 showed significant upregulation under these conditions (Tilikj et al. 2025), while none of the double-ACD sHSPs were upregulated at either temperature. The limited sHSP transcriptional response to BPA is noteworthy given that small heat shock proteins are generally considered early and sensitive indicators of cellular stress (Feder and Hofmann 1999). Their absence of strong induction may suggest that the exposure conditions applied here did not reach the threshold for a generalized transcriptional stress response. Novo et al. (2018) found that longer-term exposure (48 h) to BPA affects an earthworm’s detoxification and endocrine systems. However, it is still unclear whether sHSPs play a role under these same conditions..

**Fig. 4:**
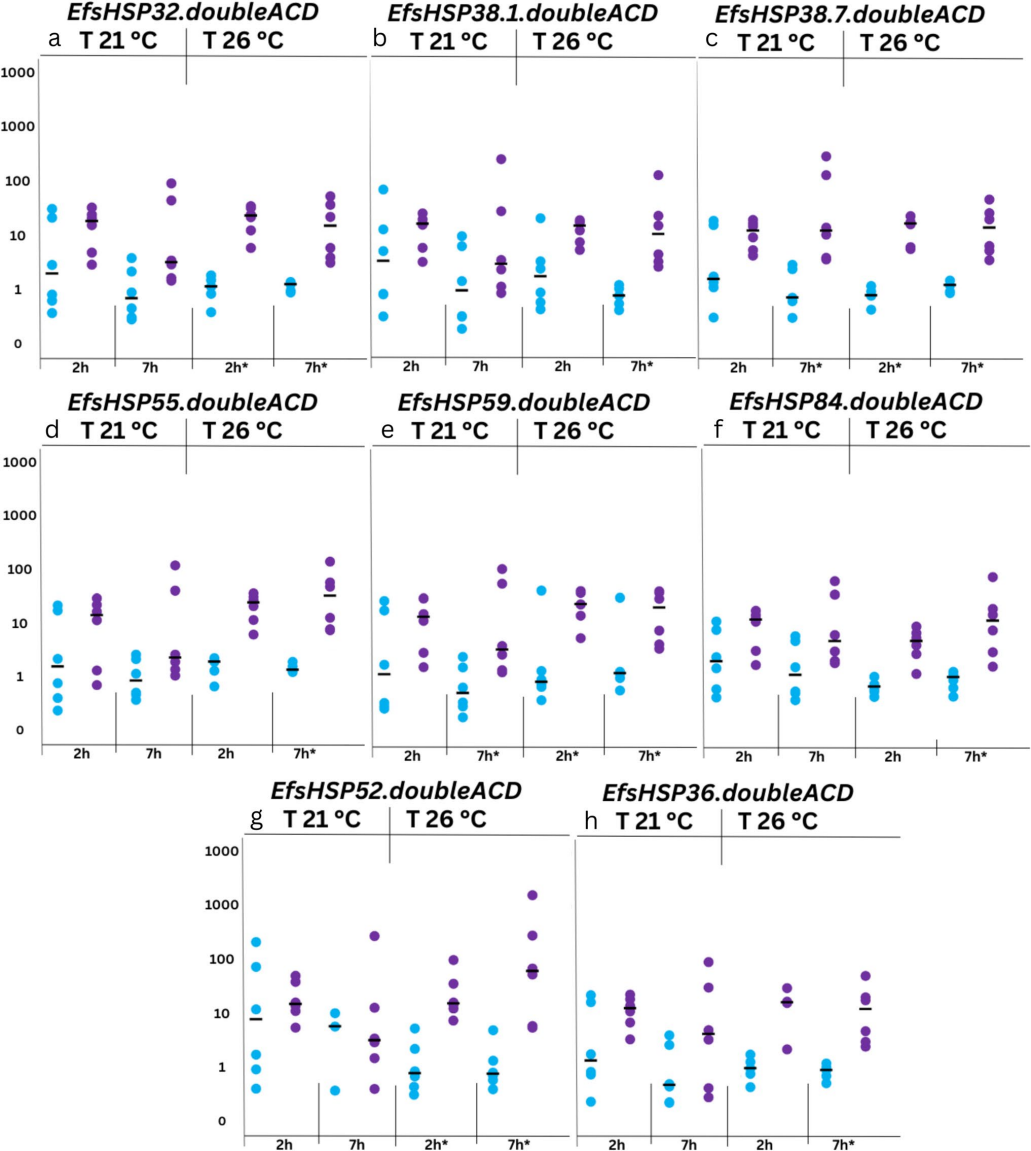
Expression profiles of *EfsHSP* genes with dual ACD domain architecture under chemical stress from endosulfan at two temperature conditions: 21 (optimal) and 26 °C (elevated, combined stress). The expression levels were measured at different time points (2 h and 7 h). Asterisks represent significant differences (*p* < 0.05) between control and exposure. Numbers on *y* axis are arbitrary

Under END exposure, combined stress led to significant upregulation of sHSPs with double-ACDs, paralleling the response observed in monomeric sHSPs. Notably, two double-ACD sHSPs, EfsHSP38.7.doubleACD and EfsHSP59.doubleACD, were significantly upregulated after 7 h at 21 °C under endosulfan exposure (Fig. 4). SmartBLAST analysis returned best matches to HSPB-family proteins, supporting classification as sHSPs (Supplementary File S2). For double-ACD proteins, query coverage is expected to be reduced when compared against single-ACD HSPBs as only one ACD aligns strongly at a time. Accordingly, these matches are interpreted as ACD-level homology rather than whole-protein equivalence, so functional equivalence cannot be inferred. A characteristic of the HSPB-family proteins is the formation of oligomeric complexes that enable interaction with diverse protein targets (Arrigo, 2013). However, oligomerization and functional properties of sHSPs containing multiple ACDs have not yet been studied. EfsHSP59.doubleACD contains a THAP domain, a feature linked to pro-apoptotic regulation in cells under abiotic stress (Balakrishnan et al. 2011). This characteristic may account for its delayed upregulation in response to the environmental stressors examined in this study.

Interestingly, EfsHSP68, which contains three ACD domains, was the only sHSP upregulated under both temperatures (Tilikj et al. 2025) suggesting that sHSPs with multiple ACDs may exhibit enhanced sensitivity to some types of chemical stress. This unique response highlights the potential for multi-ACD sHSPs to play specialized roles in chemical stress adaptation.

### Oligomerization and its role in stress-induced chaperone activity

Many metazoan sHSPs with monomeric architecture are known for their ability to dimerize and oligomerize, forming large homo- or hetero-complexes (Tedesco et al. 2022). Under normal conditions, these sHSP oligomers remain stable; however, stress triggers rapid subunit exchange, leading to alterations in oligomeric states and unmasking new substrate interaction modes, thereby expanding the range of substrate binding (Boelens 2020).

Two distinct mechanisms have been proposed to explain how sHSP oligomers behave during heat stress. Eyles and Gierasch (2010) suggest that large oligomers undergo subunit exchange, forming even larger assemblies that incorporate both sHSPs and trapped unfolded proteins. In contrast, Haslbeck et al. (2005) propose that oligomers dissociate into smaller units, such as dimers or tetramers, which then bind unfolded proteins. Despite these differences, both models emphasize the dynamic nature and structural flexibility of sHSPs, key features that underpin their chaperone activity and ability to interact with diverse substrates.

In the present study, we predicted single-chain structures only and did not model oligomeric assemblies directly; therefore, any discussion of oligomerization is hypothesis-driven and based on conserved ACD features and analogy with known sHSP systems. In this context, the delayed induction of double-ACD sHSPs under heat, desiccation, and chemical stress, relative to the early induction of monomeric sHSPs (Tilikj et al. 2025), may reflect structural differences between these two groups and is consistent with a tiered activation model. The early and robust response of monomeric sHSPs may be attributed to their inherent flexibility, which allows them to rapidly oligomerize as homo- or heterodimers under stress. In contrast, the presence of two ACD regions may introduce structural rigidity, reducing their ability to oligomerize rapidly or to undergo dynamic subunit exchange. Additionally, because many sHSPs preferentially form heterooligomers (e.g., within the HSPB family; Boelens 2020), a dual-ACD architecture could add structural complexity that reduces heterooligomerization efficiency.

The number of ACD regions also appears to influence transcriptional dynamics. Studies on genes with one, three, and seven ACDs report earlier peaks for mono-ACD genes (6 h) compared to those with three or seven ACDs (12 h; Zhang et al. 2023). Our findings, in conjunction with those by Zhang et al. (2023), indicate a fast response from the single-ACD sHSPs while sHSPs containing multiple ACDs show slower activation kinetics. By complementing the rapid, early action of single-ACD sHSPs, double-ACD sHSPs help establish a sustained chaperone network that is vital for survival under prolonged or severe stress conditions. In ecological terms, such sequential recruitment may be advantageous in soils, where earthworms experience fluctuating temperature and moisture regimes and stress episodes can be prolonged or repeated.

## Conclusions

This study highlights the complexity and diversity of small heat shock proteins (sHSPs) with two alpha-crystallin domains (ACDs) in *Eisenia fetida.* These double-ACD sHSPs exhibit stimulus-specific transcriptional responses under heat, desiccation, and chemical stress conditions. The delayed transcriptional activation, compared to their monomeric counterparts, suggests a complementary role in stress adaptation, likely in long-term cellular protection or through an alternative mechanism of action. Distinct, stressor-specific activation of double-ACD sHSPs under combined heat and chemical exposure highlights their potential as molecular indicators of earthworm resilience and sublethal stress, supporting ecological risk assessment and soil biomonitoring in contaminated and climate-impacted environments. Importantly, these combined stress treatments reflect the co-occurring chemical and abiotic pressures typical of soil environments. Future work should aim to elucidate the oligomerization behavior and substrate interactions of these proteins to better understand their contribution to stress tolerance.

## Supplementary Information

Below is the link to the electronic supplementary material.

ESM1 (DOCX 7.44 MB)

ESM2 (XLSX 239 KB)

## Data Availability

Data available on request from the authors.
