# Supplementary material for: Small heat shock proteins with two alpha-crystallin domains: a new set of proteins in the earthworm Eisenia fetida with differential transcriptional responses to stressors
Source: Environ Sci Pollut Res Int. 2026 May 11;33(17):8095–110. doi: 10.1007/s11356-026-37811-y (PMC13226371; doi:10.1007/s11356-026-37811-y)
Supplement: Supplementary file 1 — (DOCX 7.44 MB) [file 11356_2026_37811_MOESM1_ESM.docx]

# **Dataset S1**

# **AlphaFold predicted models**

Structural predictions generated using AlphaFold 2.1, via ChimeraX version 1.8 (2024-06-10) and ColaFold, using default options and incorporating PDB templates for structure prediction (Goddard et al., 2018; Pettersen et al., 2021; Mirdita et al., 2022).

## **EfsHSP68 (triple-ACD)**

**Sequence and secondary structure**


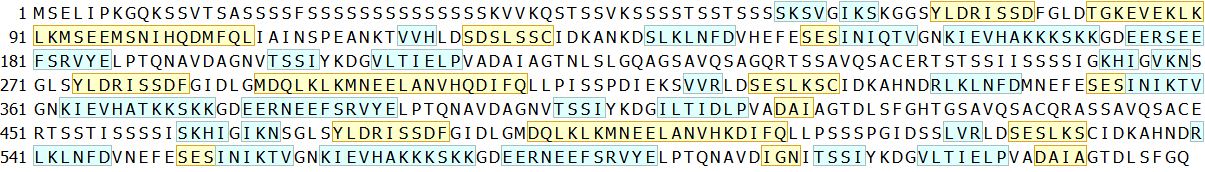


**3D model**

| 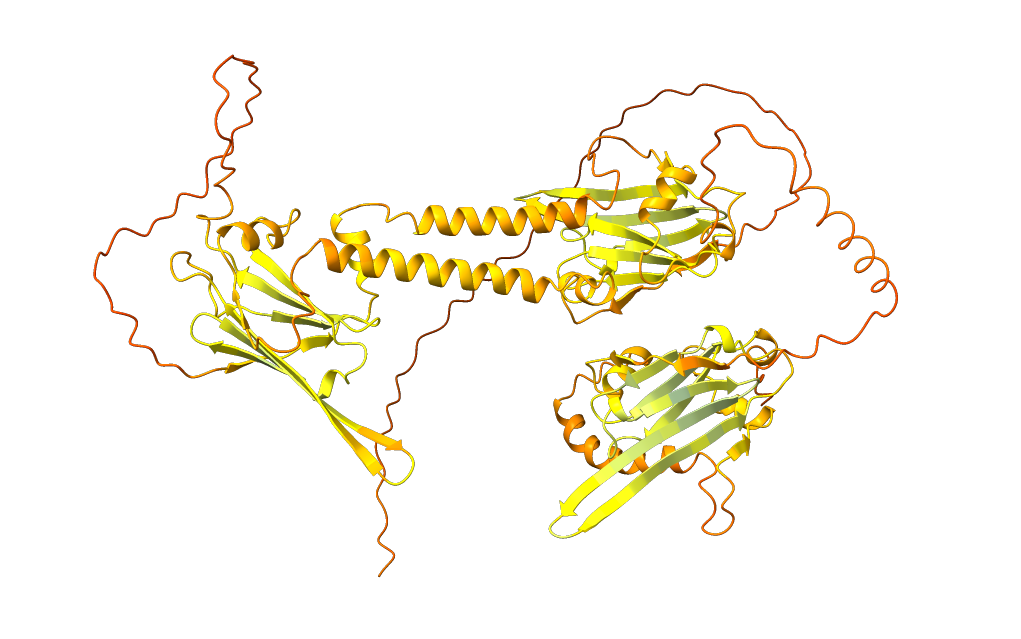 | 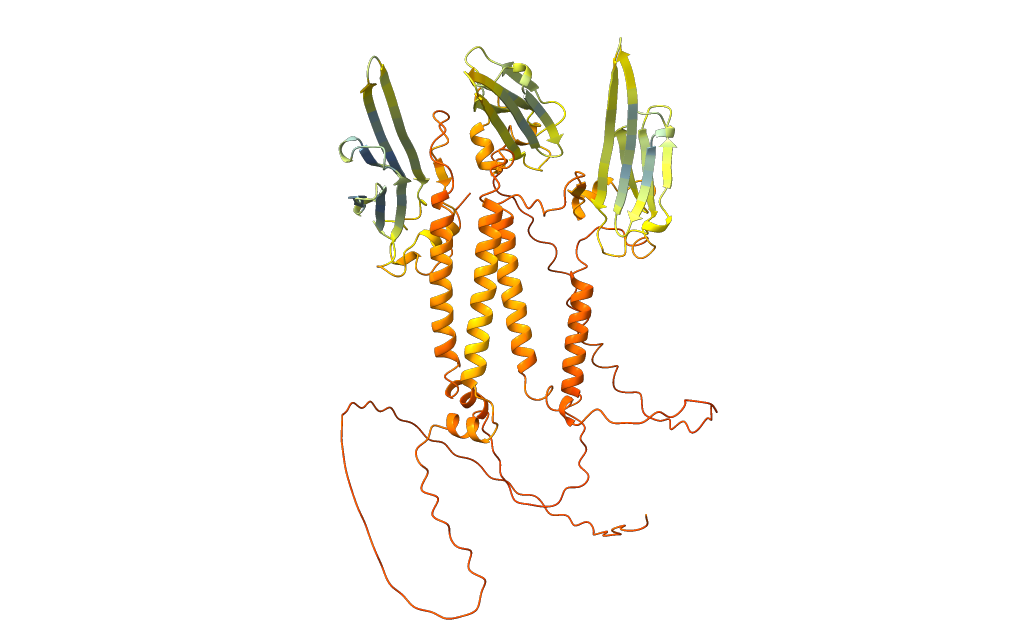 |
| --- | --- |
| Best model: rank 01/05 | Rank 03/05 |

| Per-residue model confidence score (pLDDT) between 0 and 100: | |
| --- | --- |
| Very high (pLDDT > 90)  High (90 > pLDDT > 70)  Low (70 > pLDDT > 50)  Very low (pLDDT < 50) |  |

**PAE plots for AlphaFold’s five predicted models**

**
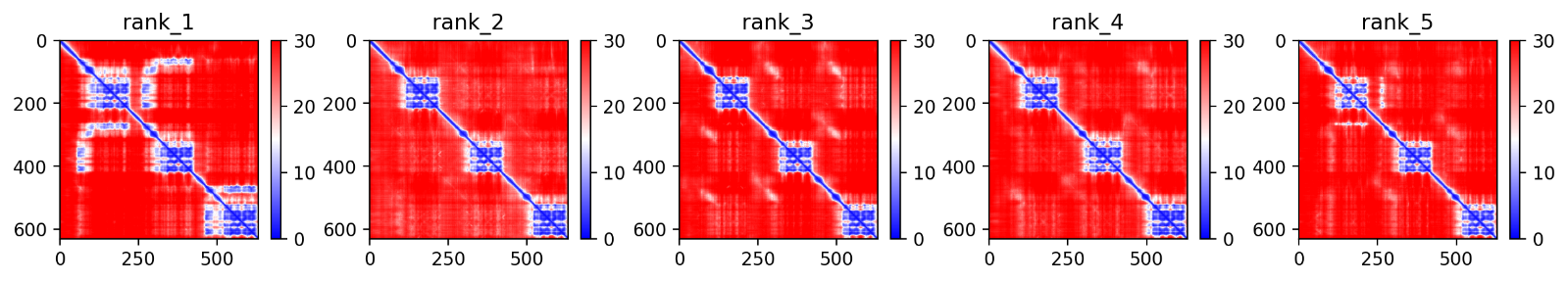
**

## **EfsHSP38.7.doubleACD (double-ACD)**

**Sequence and secondary structure**


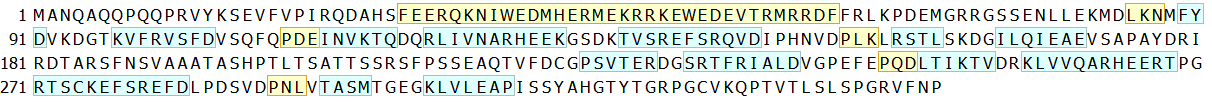


**3D model**

| 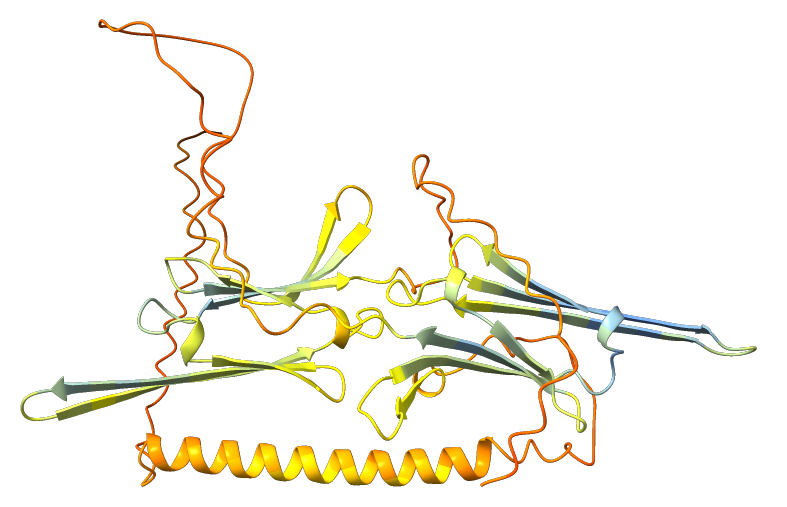 | 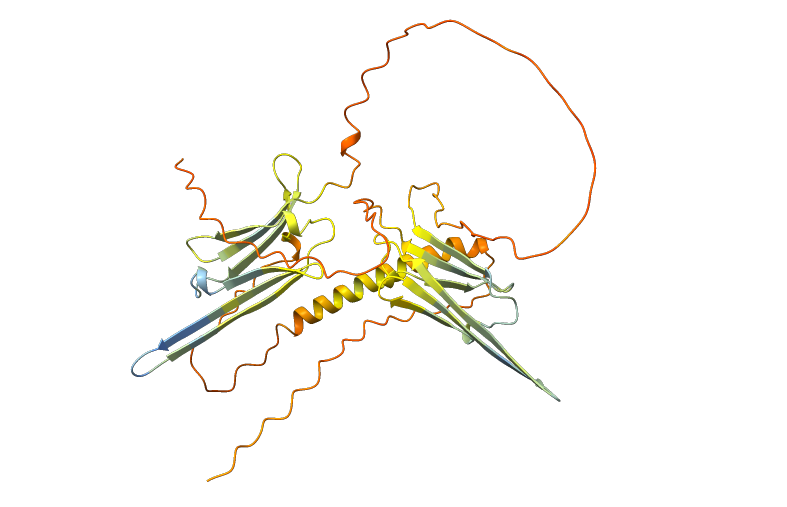 |
| --- | --- |
| Best model: rank 01/05 | Rank 03/05 |

| Per-residue model confidence score (pLDDT) between 0 and 100: | |
| --- | --- |
| Very high (pLDDT > 90)  High (90 > pLDDT > 70)  Low (70 > pLDDT > 50)  Very low (pLDDT < 50) |  |

**PAE plots for AlphaFold’s five predicted models**

**
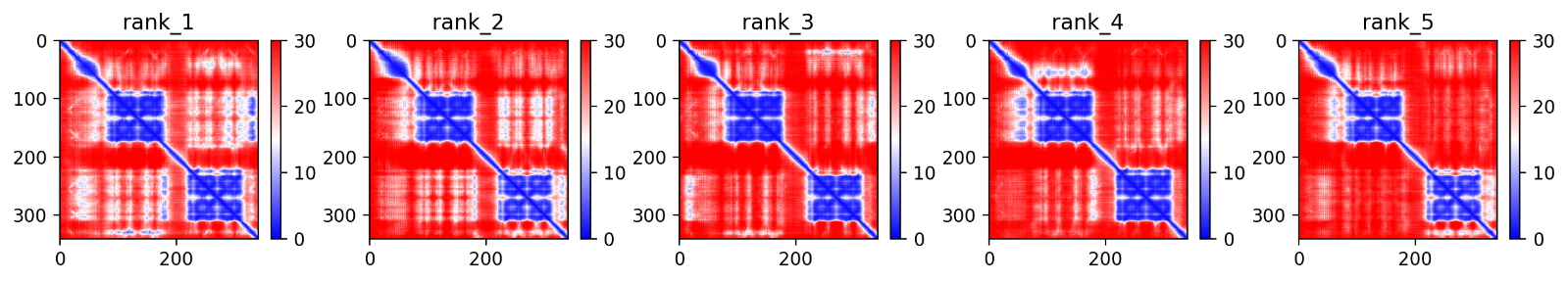
**

## **EfsHSP55.doubleACD (double-ACD)**

**Sequence and secondary structure**


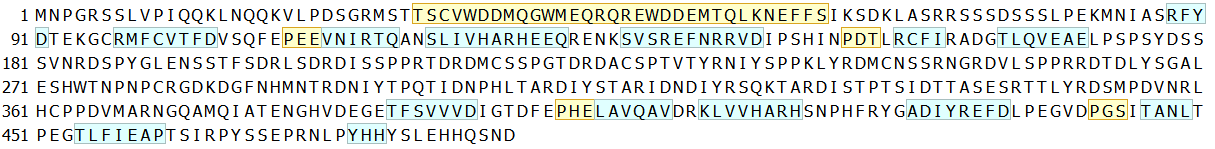


**3D model**

| 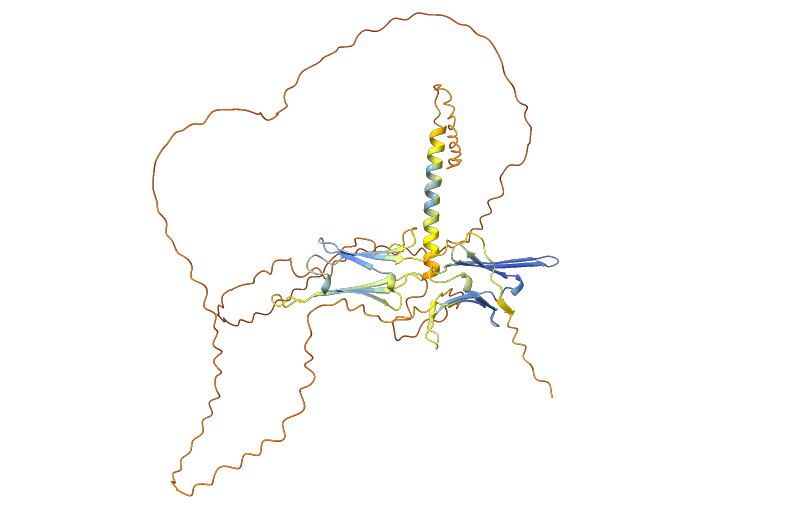 | **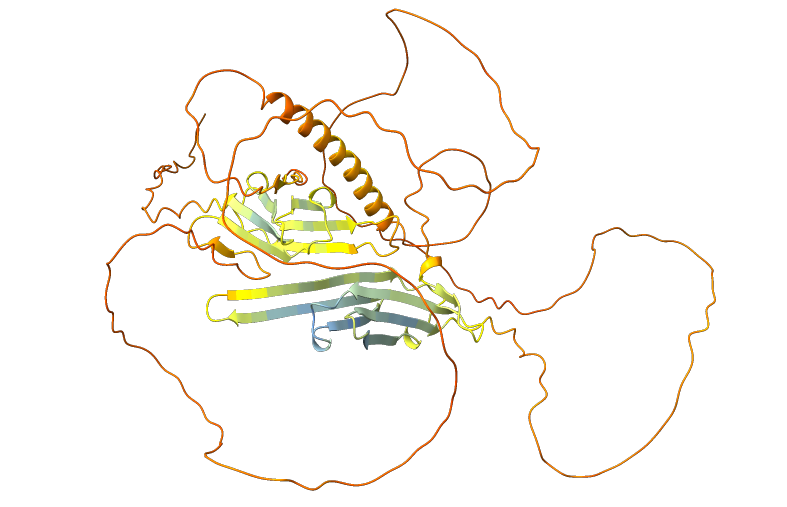** |
| --- | --- |
| Best model: rank 01/05 | Rank 02/05 |

| Per-residue model confidence score (pLDDT) between 0 and 100: | |
| --- | --- |
| Very high (pLDDT > 90)  High (90 > pLDDT > 70)  Low (70 > pLDDT > 50)  Very low (pLDDT < 50) |  |

**PAE plots for AlphaFold’s five predicted models**


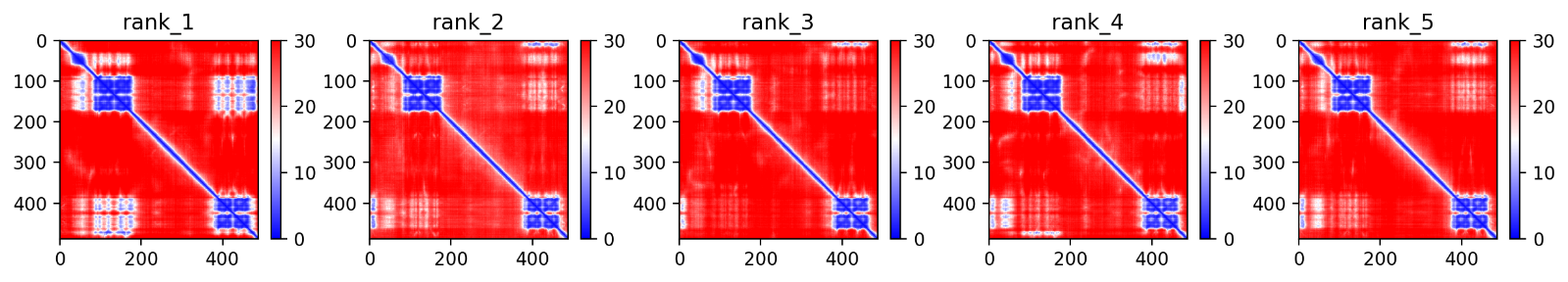


## **EfsHSP38.1.doubleACD (double-ACD)**

**Sequence and secondary structure**


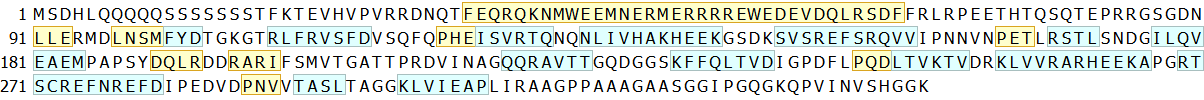


**3D model**

| 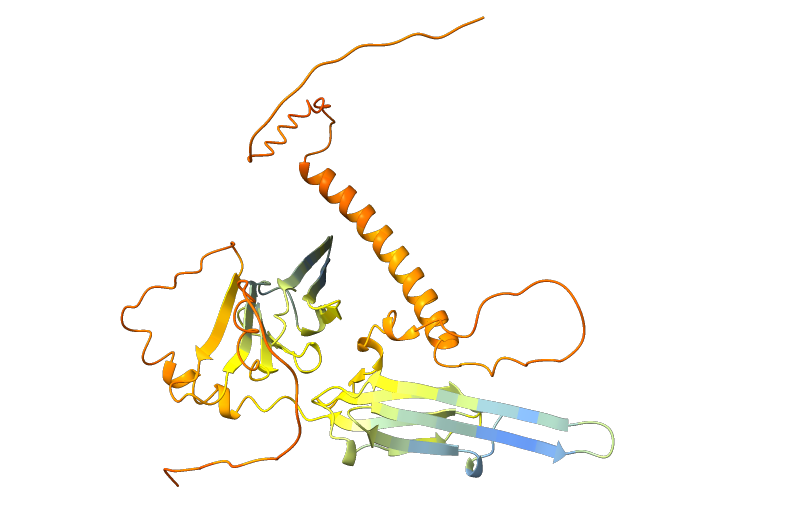 | 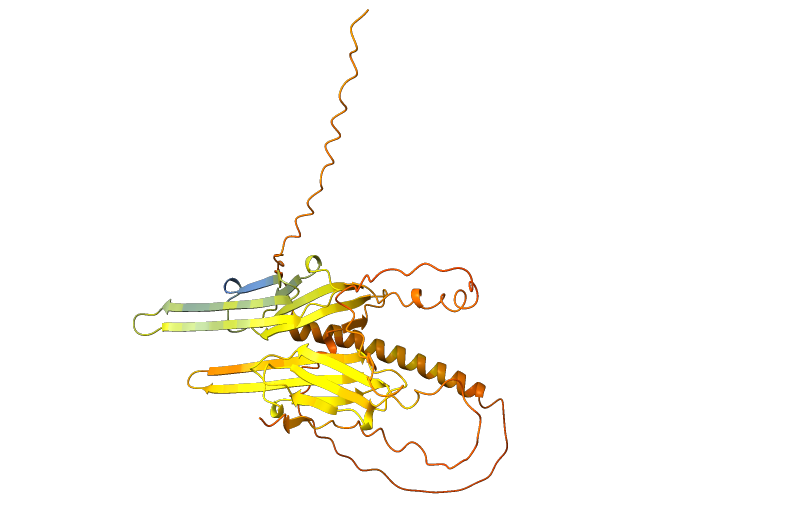 |
| --- | --- |
| Best model: rank 01/05 | Rank 04/05 |

| Per-residue model confidence score (pLDDT) between 0 and 100: | |
| --- | --- |
| Very high (pLDDT > 90)  High (90 > pLDDT > 70)  Low (70 > pLDDT > 50)  Very low (pLDDT < 50) |  |

**PAE plots for AlphaFold’s five predicted models**


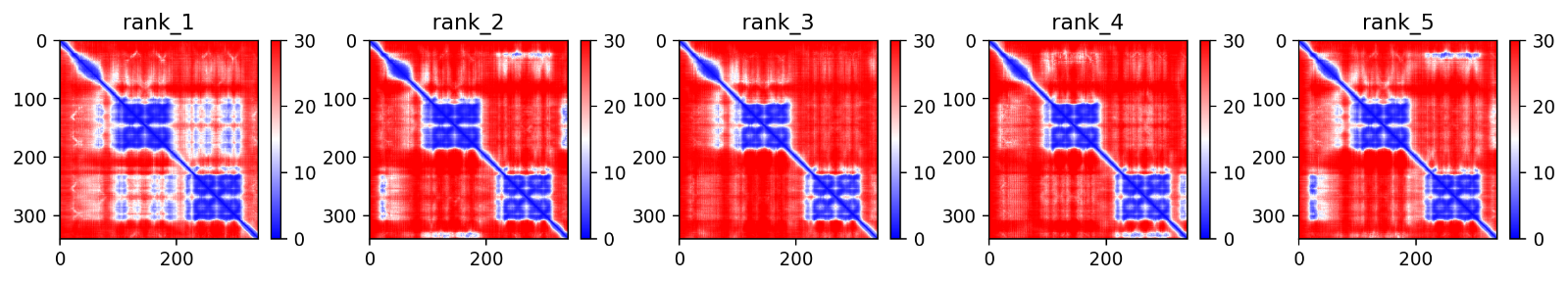


## **EfsHSP36.doubleACD (double-ACD)**

**Sequence and secondary structure**


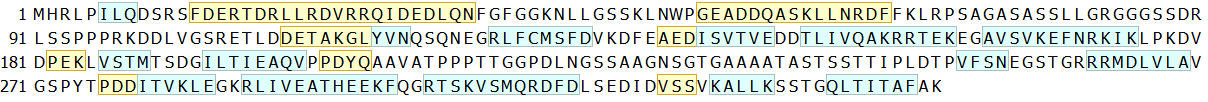


**3D model**

| 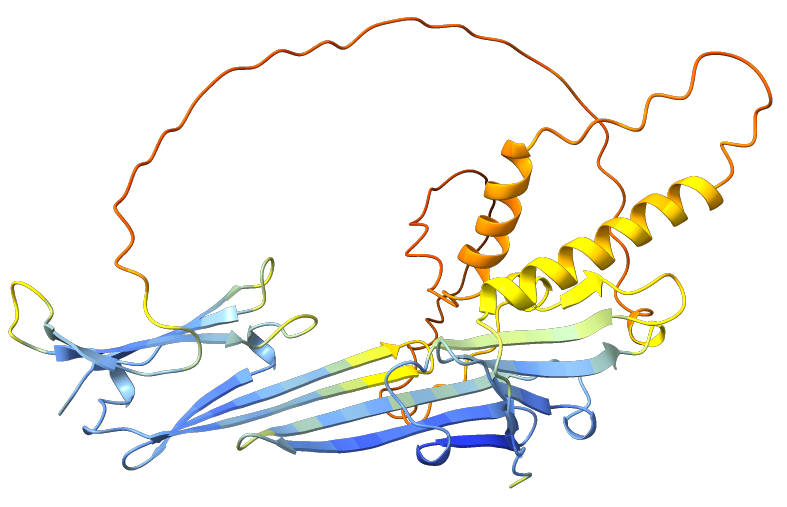 | **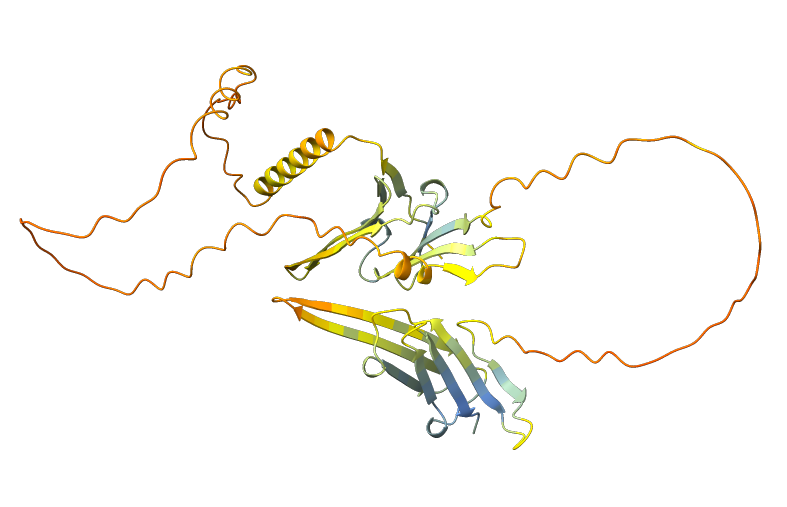** |
| --- | --- |
| Best model: rank 01/05 | Rank 03/05 |

| Per-residue model confidence score (pLDDT) between 0 and 100: | |
| --- | --- |
| Very high (pLDDT > 90)  High (90 > pLDDT > 70)  Low (70 > pLDDT > 50)  Very low (pLDDT < 50) |  |

**PAE plots for AlphaFold’s five predicted models**


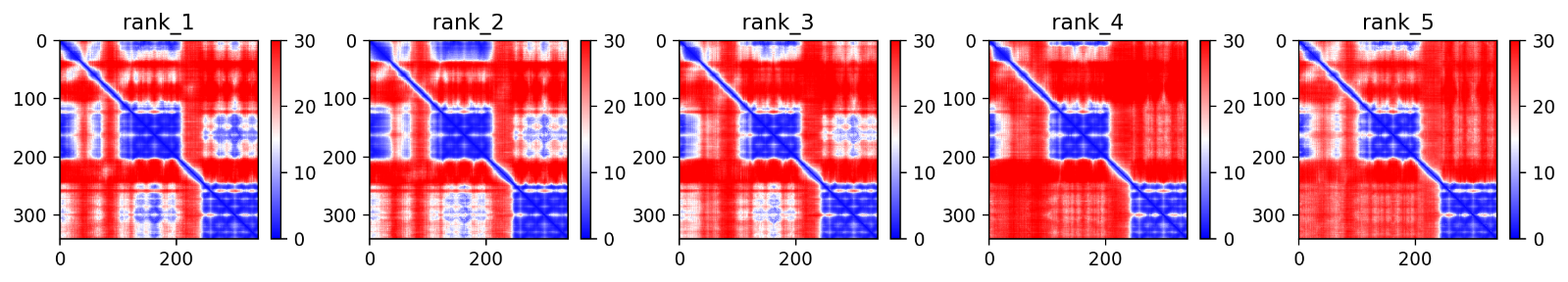


## **EfsHSP59.doubleACD (double-ACD)**

THAP DOMAIN

**Sequence and secondary structure**


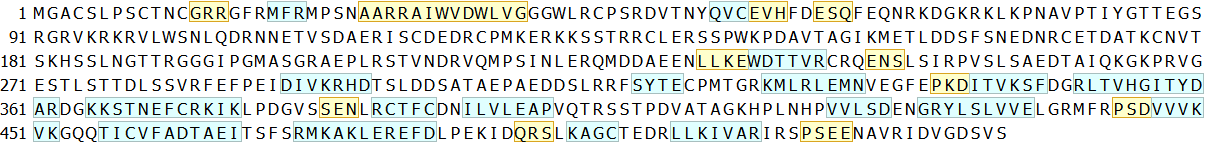


**3D model**

| 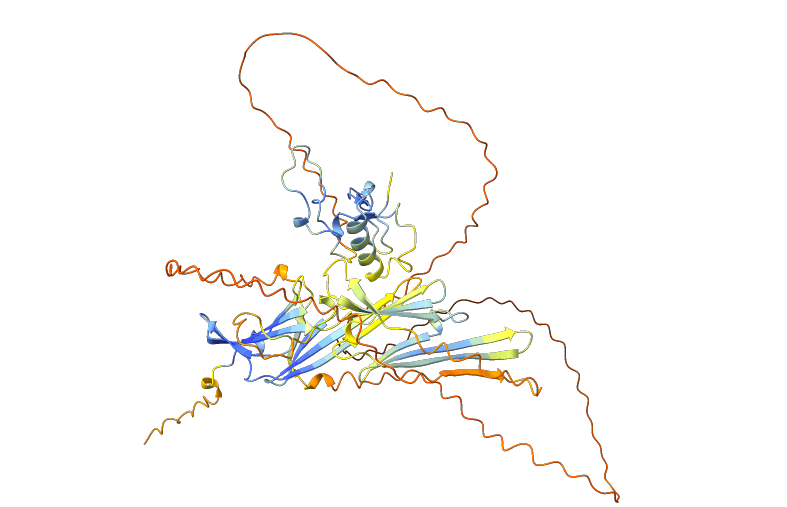 | 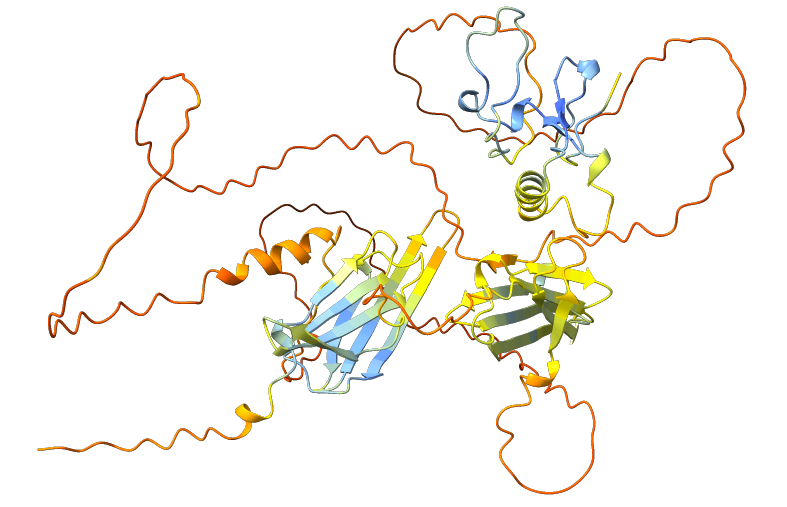 |
| --- | --- |
| Best model: rank 01/05 | Rank 03/05 |

| Per-residue model confidence score (pLDDT) between 0 and 100: | |
| --- | --- |
| Very high (pLDDT > 90)  High (90 > pLDDT > 70)  Low (70 > pLDDT > 50)  Very low (pLDDT < 50) |  |

**PAE plots for AlphaFold’s five predicted models**


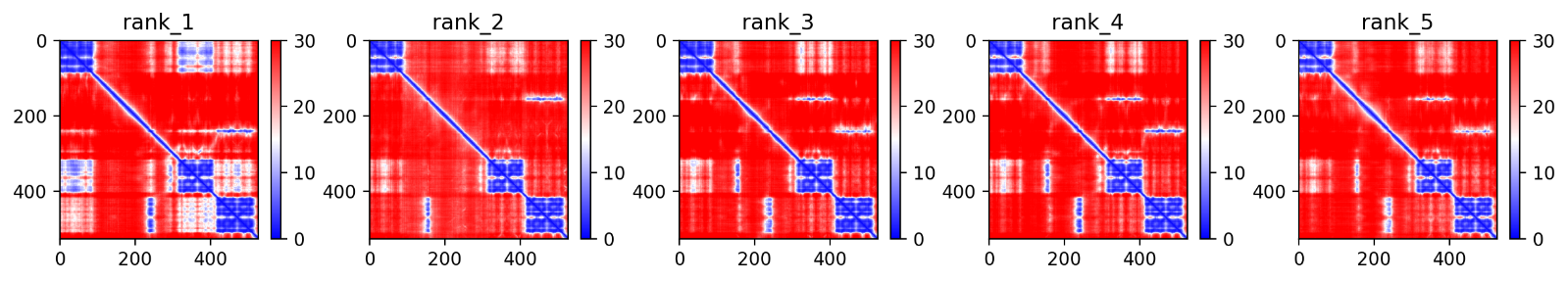


## **EfsHSP32.doubleACD (double-ACD)**

**Sequence and secondary structure**


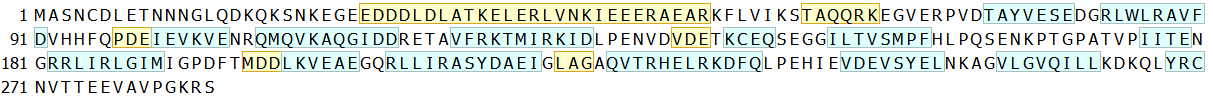


**3D model**

| 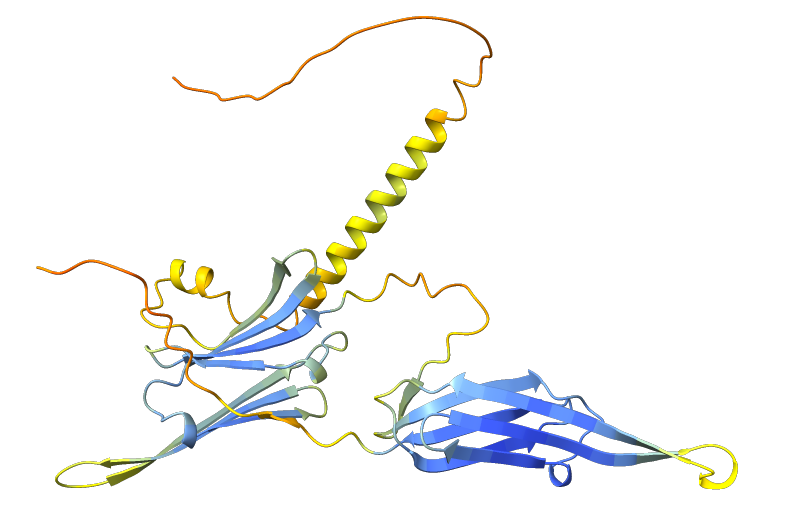 |  |
| --- | --- |
| Best model: rank 01/05 |  |
| **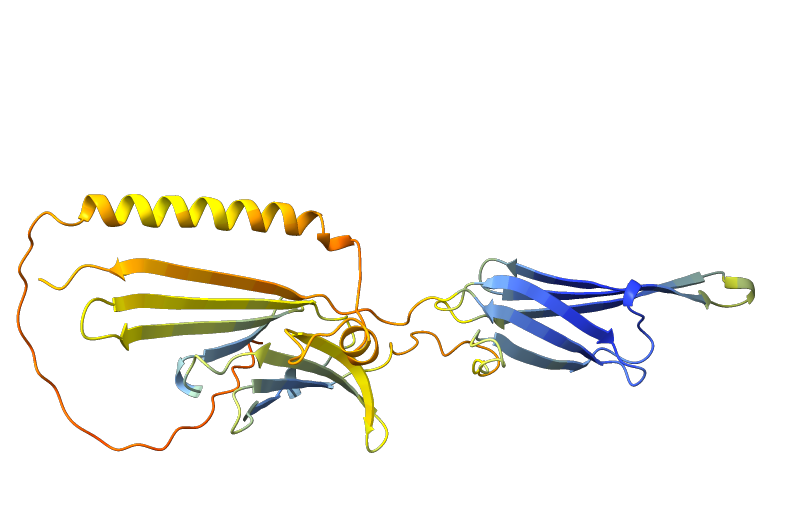** |  |
| Rank 04/05 |  |

| Per-residue model confidence score (pLDDT) between 0 and 100: | |
| --- | --- |
| Very high (pLDDT > 90)  High (90 > pLDDT > 70)  Low (70 > pLDDT > 50)  Very low (pLDDT < 50) |  |

**PAE plots for AlphaFold’s five predicted models**


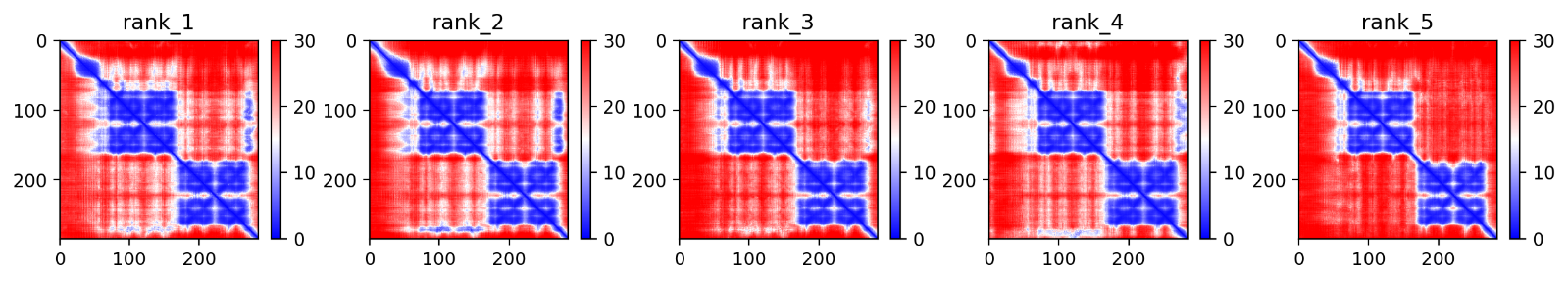


## **EfsHSP33.doubleACD (double-ACD)**

**Sequence and secondary structure**


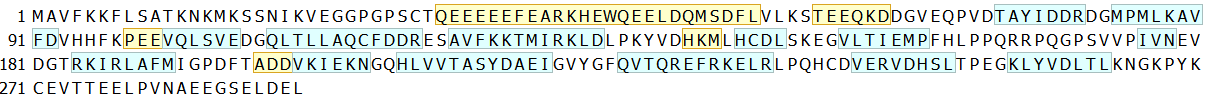


**3D model**

| 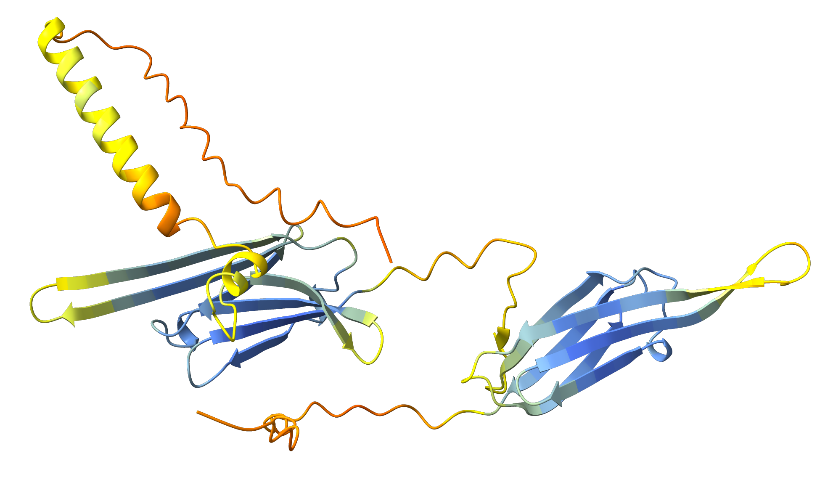 | 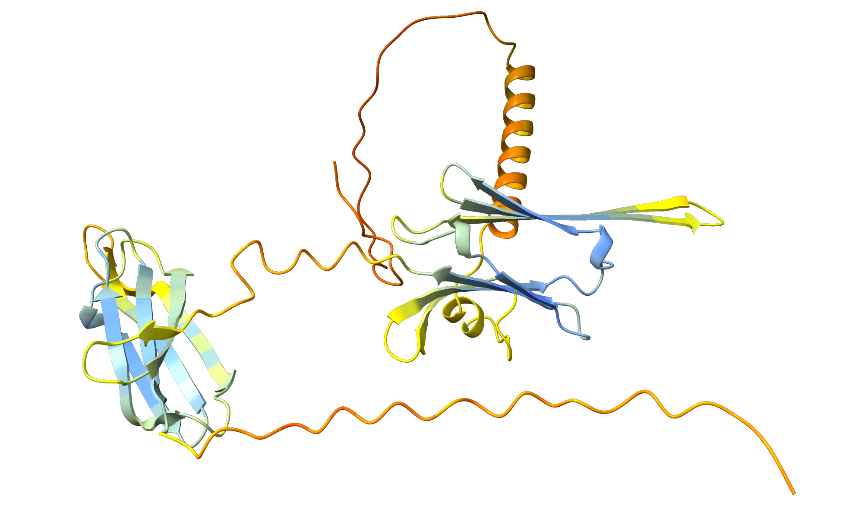 |
| --- | --- |
| Best model: rank 01/05 | Rank 02/05 |

| Per-residue model confidence score (pLDDT) between 0 and 100: | |
| --- | --- |
| Very high (pLDDT > 90)  High (90 > pLDDT > 70)  Low (70 > pLDDT > 50)  Very low (pLDDT < 50) |  |

**PAE plots for AlphaFold’s five predicted models**


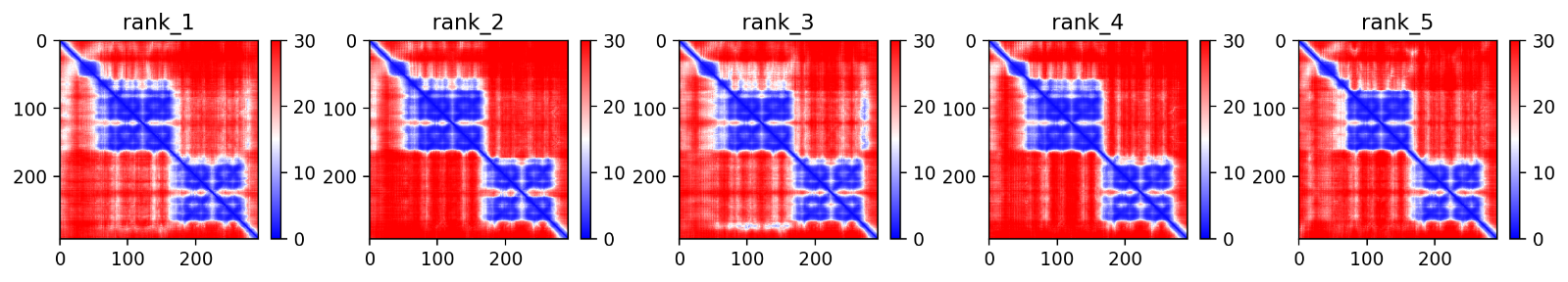


## **EfsHSP84.doubleACD (double-ACD)**

**Sequence and secondary structure**


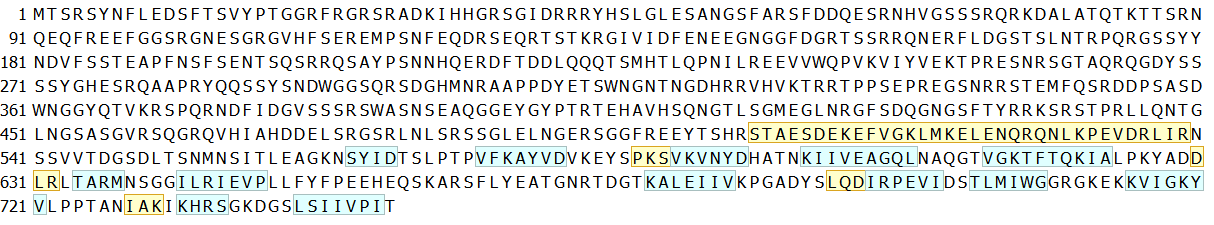


**3D model**

| 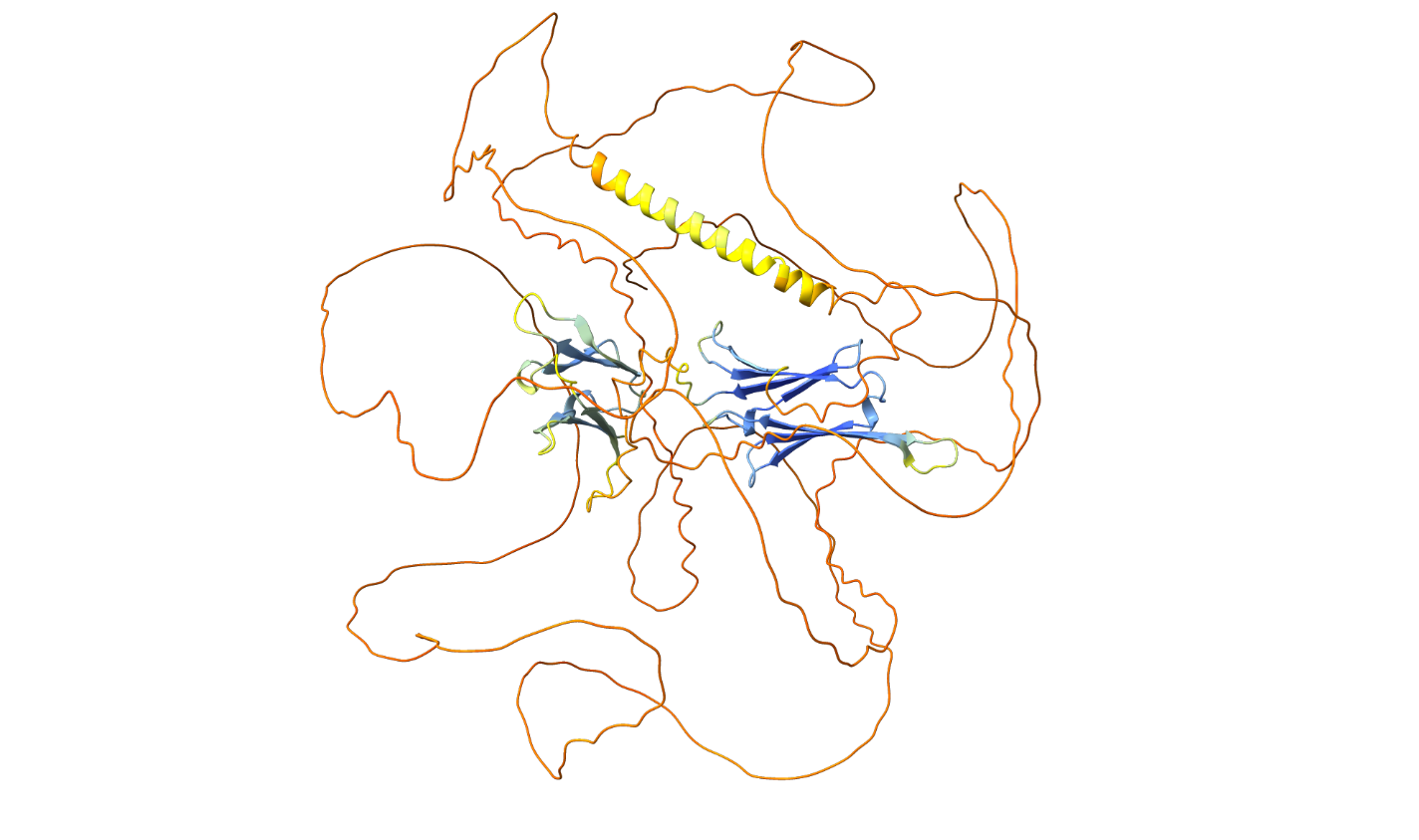 | 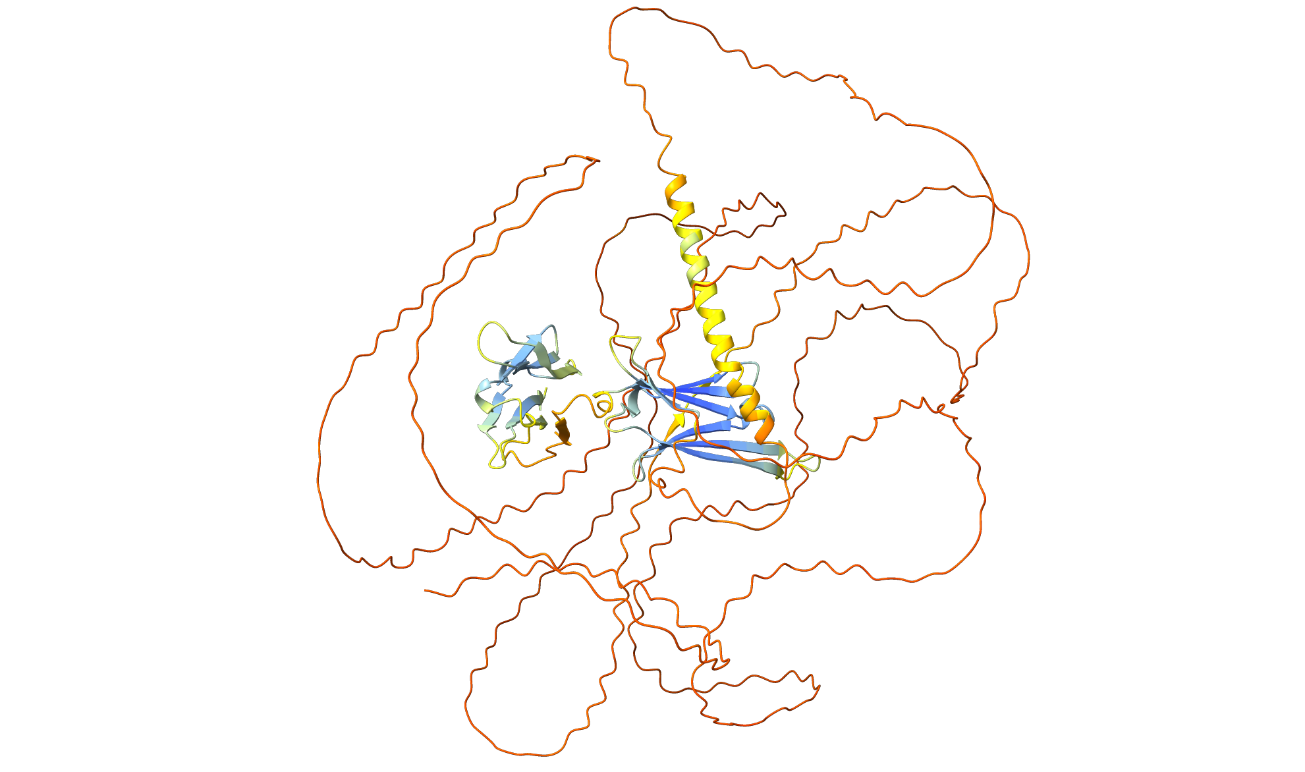 |
| --- | --- |
| Best model: rank 01/05 | Rank 05/05 |

| Per-residue model confidence score (pLDDT) between 0 and 100: | |
| --- | --- |
| Very high (pLDDT > 90)  High (90 > pLDDT > 70)  Low (70 > pLDDT > 50)  Very low (pLDDT < 50) |  |

**PAE plots for AlphaFold’s five predicted models**

**
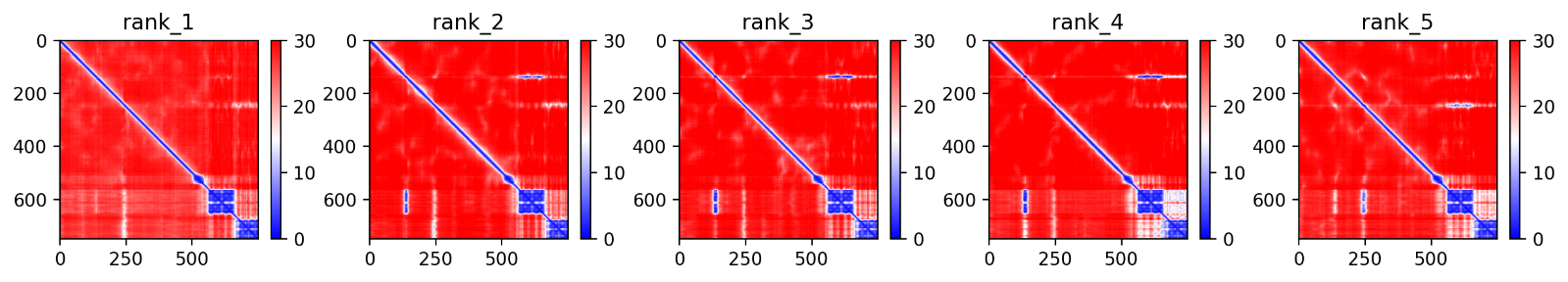
**

## **EfsHSP52.doubleACD (double-ACD)**

**Sequence and secondary structure**


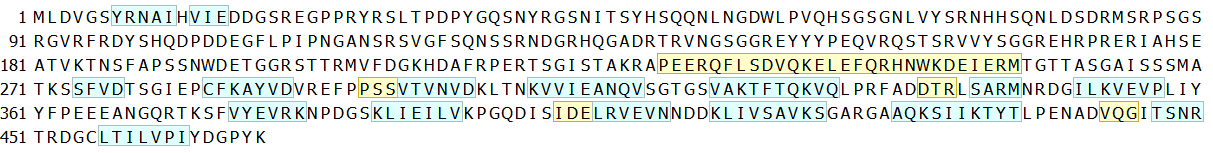


**3D model**

| 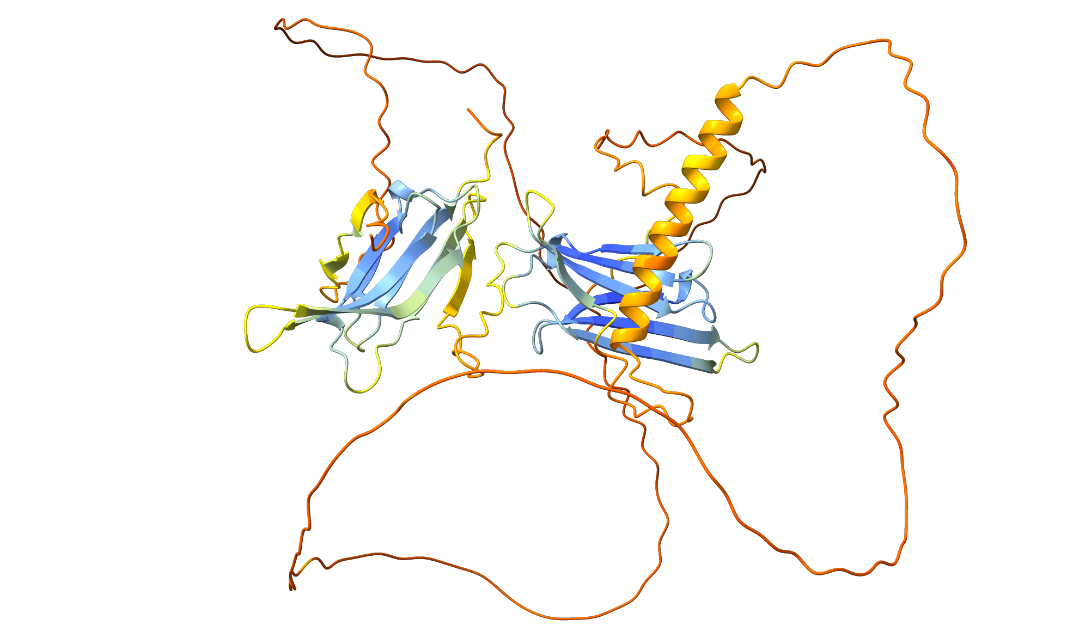 | 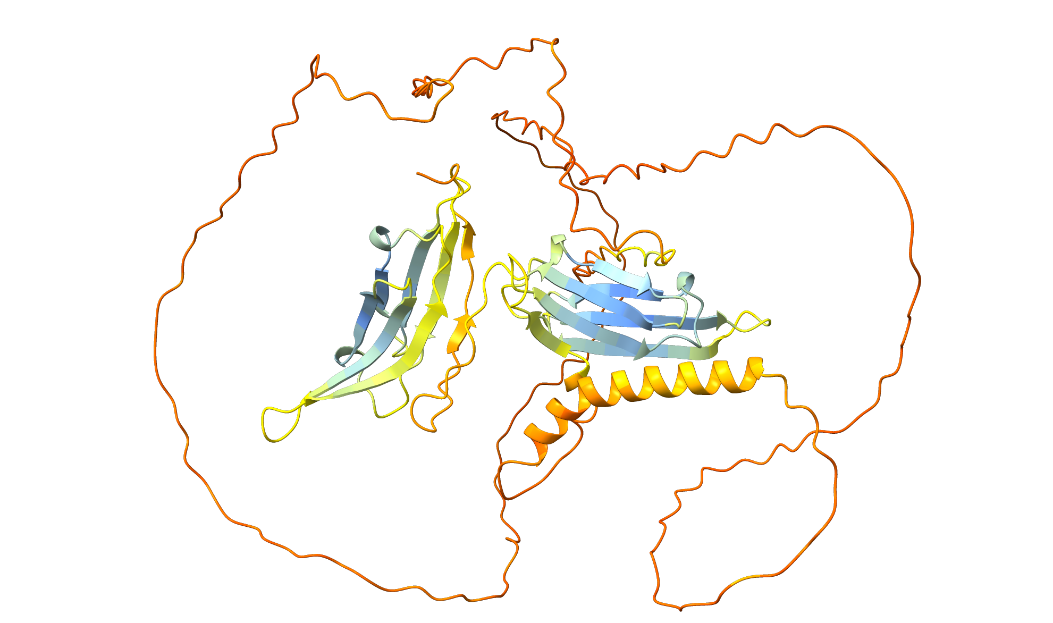 |
| --- | --- |
| Best model: rank 01/05 | Rank 04/05 |

| Per-residue model confidence score (pLDDT) between 0 and 100: | |
| --- | --- |
| Very high (pLDDT > 90)  High (90 > pLDDT > 70)  Low (70 > pLDDT > 50)  Very low (pLDDT < 50) |  |

**PAE plots for AlphaFold’s five predicted models**


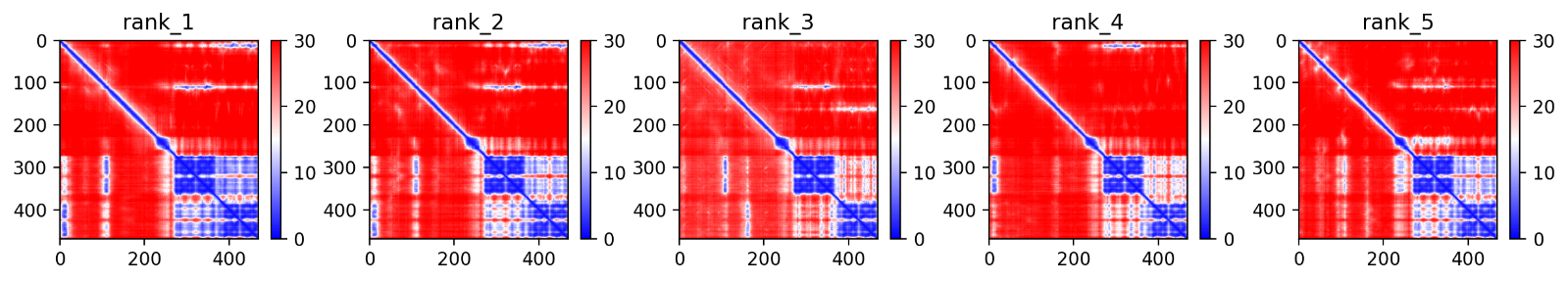


# **Structures of other homologous proteins**

Structures of homologous proteins identified through SmartBLAST. These structure were obtained, via UniProt (The UniProt Consortium, 2023) from AlphaFold DB (Jumper et al., 2021; Varadi et al., 2022), and Protein Data Bank (PDBe) (Armstrong et al., 2020).

| **UniProtKB entry: P04792.** Heat shock protein beta-1 (HSPB1). *Homo sapiens* | |
| --- | --- |
| 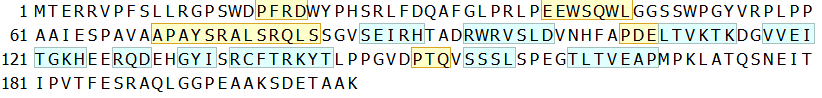  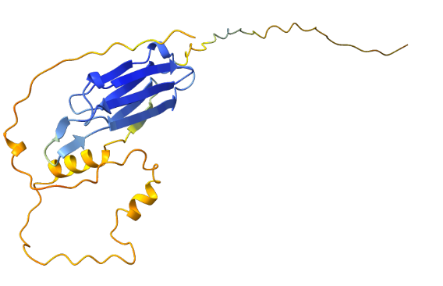  <https://alphafold.ebi.ac.uk/entry/P04792> | HSPB1 dimers  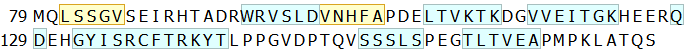  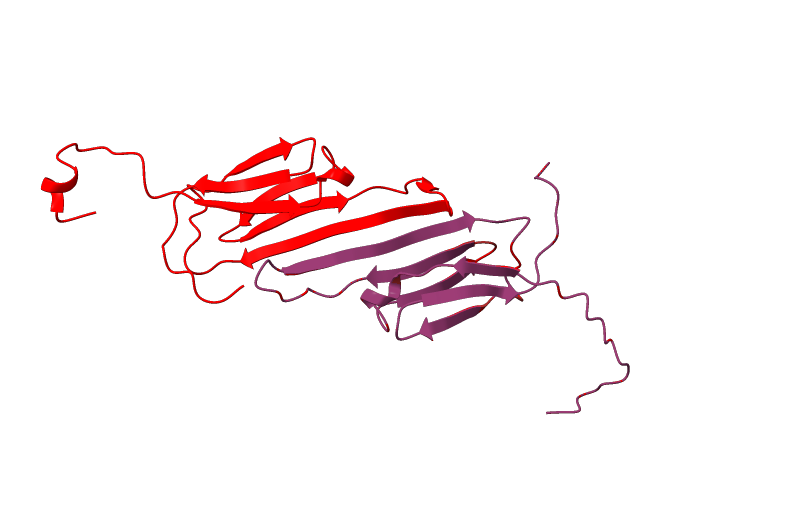  <https://www.ebi.ac.uk/pdbe-srv/view/entry/2N3J> |
| **UniProtKB entry: O14558.** Heat shock protein beta-6 (HSPB6). *Homo sapiens* | |
| 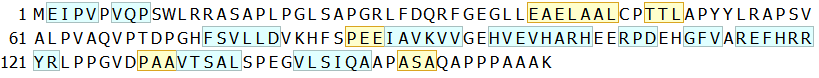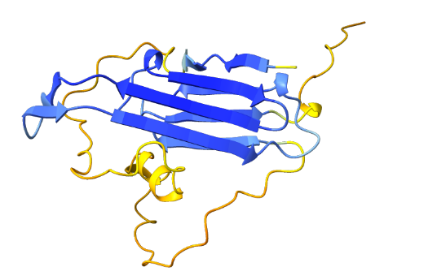  <https://alphafold.ebi.ac.uk/entry/O14558> | HSPB6 dimers / tetramers  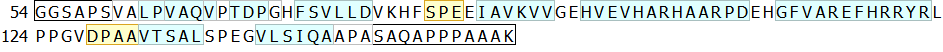  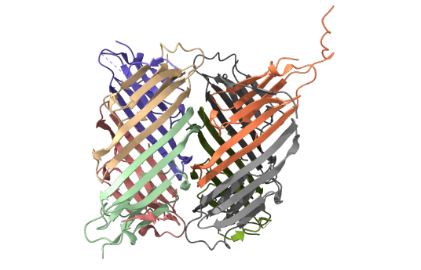  <https://www.ebi.ac.uk/pdbe-srv/view/entry/4JUT> |
| **UniProtKB entry:** Q16082  Heat shock protein beta-2 (HSPB2). *Homo sapiens* | **UniProtKB entry:** Q12988  Heat shock protein beta-3 (HSPB3). *Homo sapiens* |
| 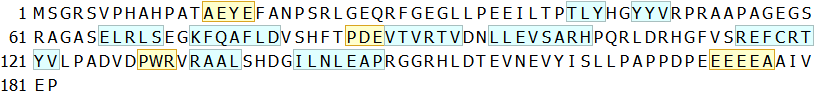  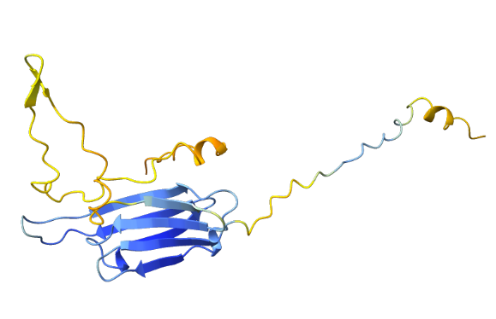  <https://alphafold.ebi.ac.uk/entry/Q16082> | 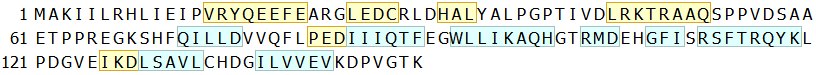  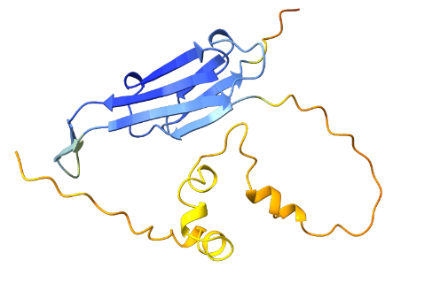  <https://alphafold.ebi.ac.uk/entry/Q12988> |
| **UniProtKB entry:** Q16082  Heterotetramen of HSPB3/HSPB2. *Homo sapiens* | **UniProtKB entry:** A0A024R3B9  Crystallin alpha B (CRYAB). *Homo sapiens* |
| 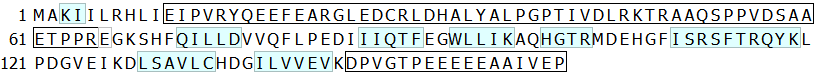  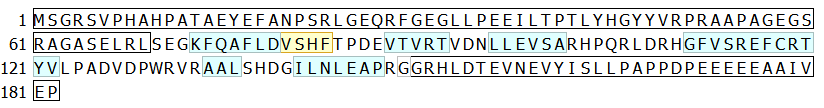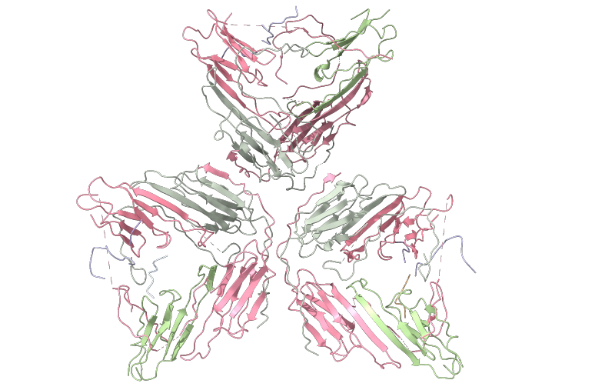  <https://www.ebi.ac.uk/pdbe-srv/view/entry/6F2R> | 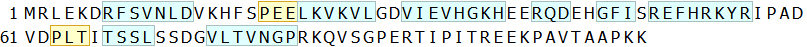  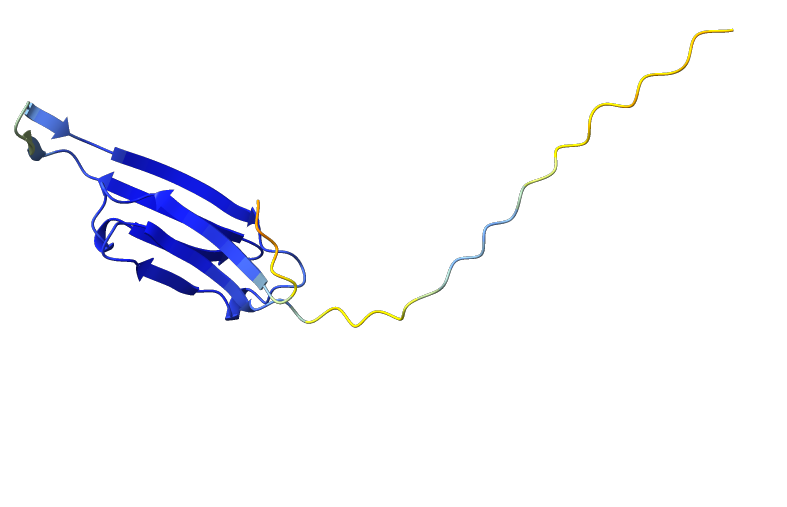  <https://alphafold.ebi.ac.uk/entry/A0A024R3B9> |
| **UniProtKB entry:** P34328  Heat shock protein Hsp-12.2. *Caenorhabditis elegans* | **UniProtKB entry:** G5EF99  SHSP domain-containing protein. *C. elegans* |
| 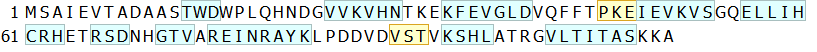  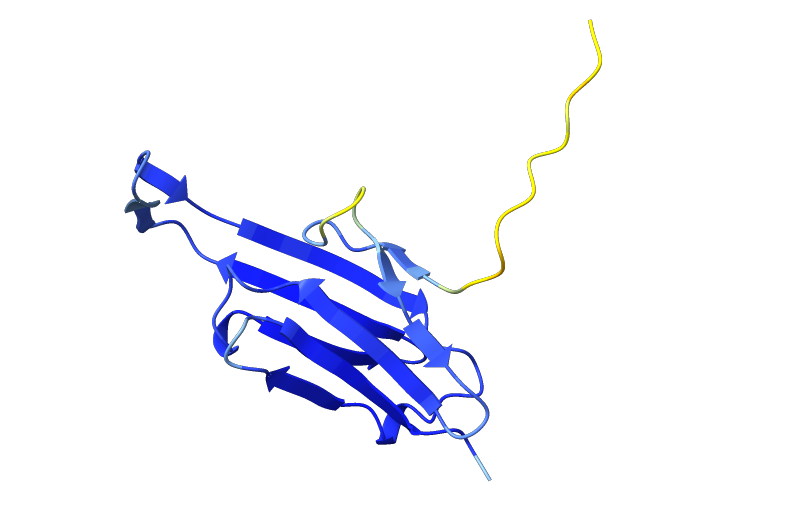  <https://alphafold.ebi.ac.uk/entry/P34328> | 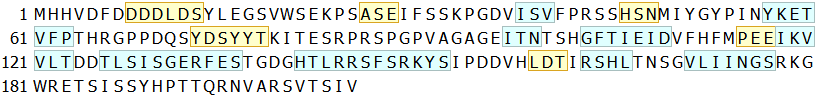  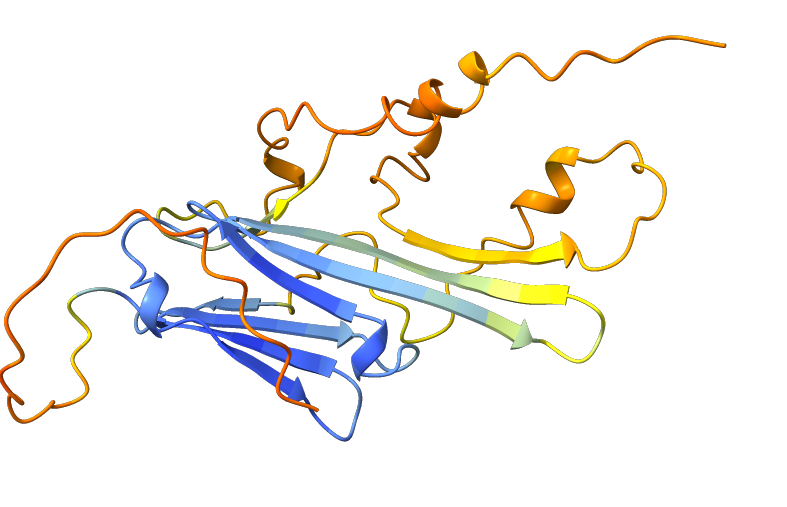<https://alphafold.ebi.ac.uk/entry/G5EF99> |
| **UniProtKB entry:** Q5H9M9  **SHSP domain-containing protein (hsp-25)**  ***Caenorhabditis elegans*** | **UniProtKB entry:** Q17849  **SHSP domain-containing protein (hsp-25)**  ***Caenorhabditis elegans*** |
| 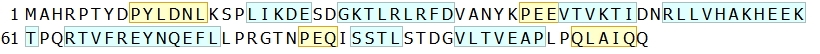  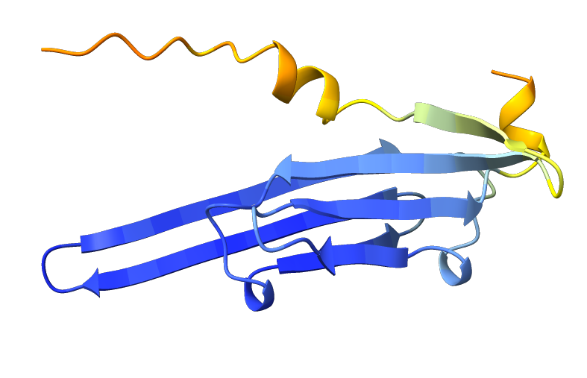  <https://alphafold.ebi.ac.uk/entry/Q5H9M9> | 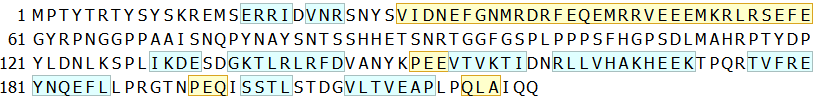  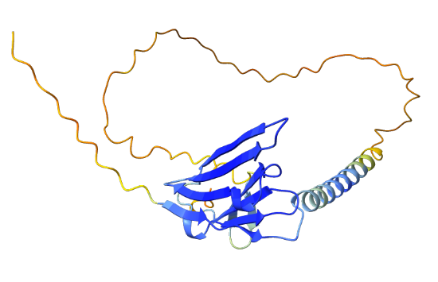  <https://alphafold.ebi.ac.uk/entry/Q17849> |
| **UniProtKB entry:** M9NHC5  Uncharacterized protein, isoform C (dHspB8)  *Drosophila melanogaster* | **UniProtKB entry:** Q9VSA9  Dmel\CG7409  *Drosophila melanogaster* |
| 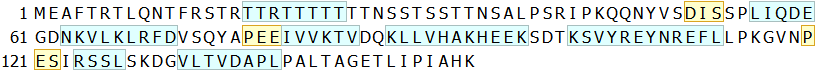  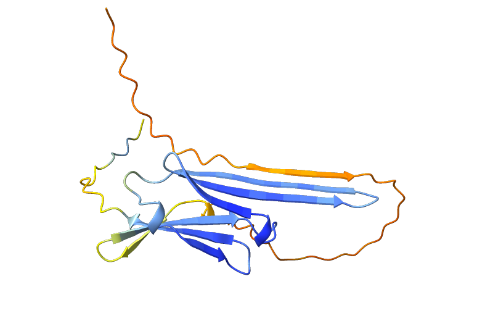  <https://alphafold.ebi.ac.uk/entry/M9NHC5> | 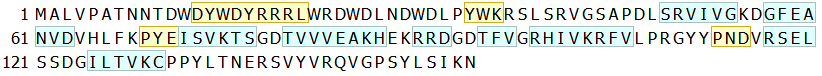  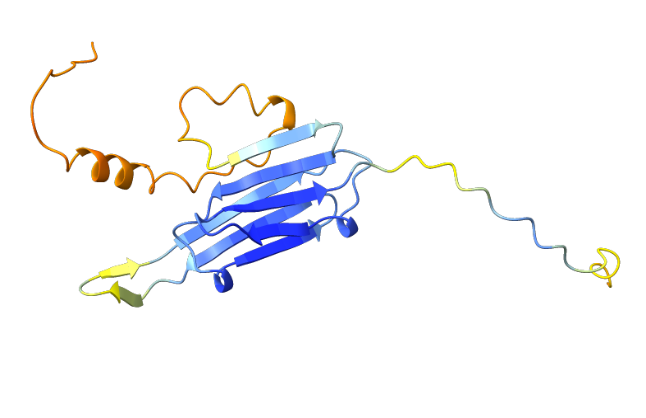  <https://alphafold.ebi.ac.uk/entry/Q9VSA9> |
| **UniProtKB entry:** P82147  Protein lethal(2)essential for life (l(2)efl)  *Drosophila melanogaster* | **UniProtKB entry:** Q9FHQ3_AtHSP15.7  15.7 kDa heat shock protein, peroxisomal (HSP15.7) *Arabidopsis thaliana* |
| 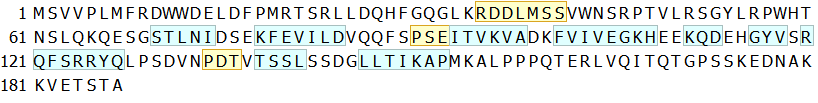  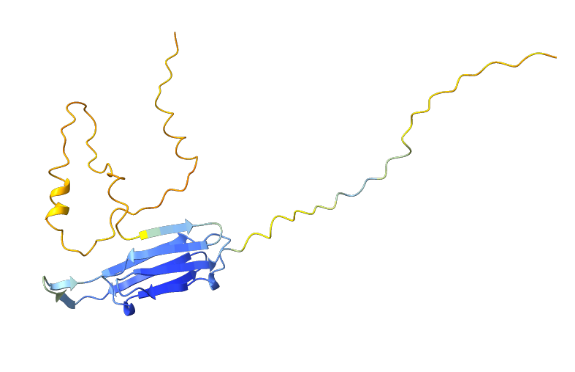  <https://alphafold.ebi.ac.uk/entry/P82147> | 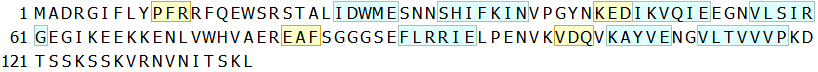  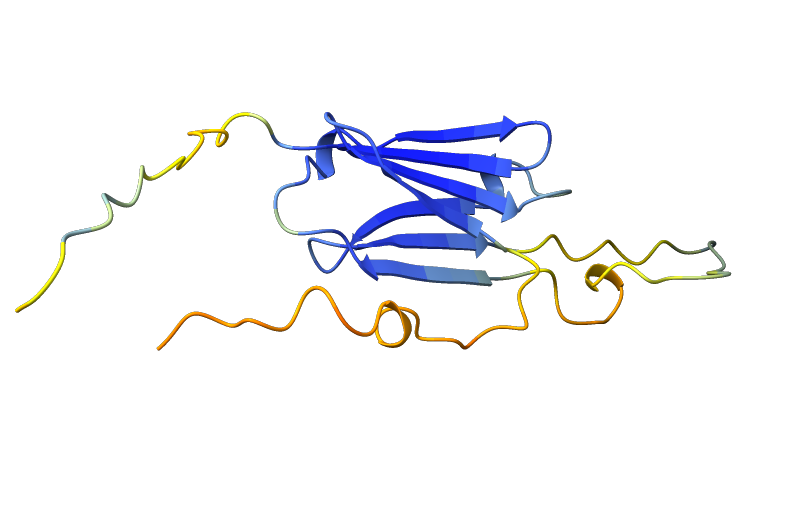  <https://alphafold.ebi.ac.uk/entry/Q9FHQ3> |

# **Supplementary Figures**


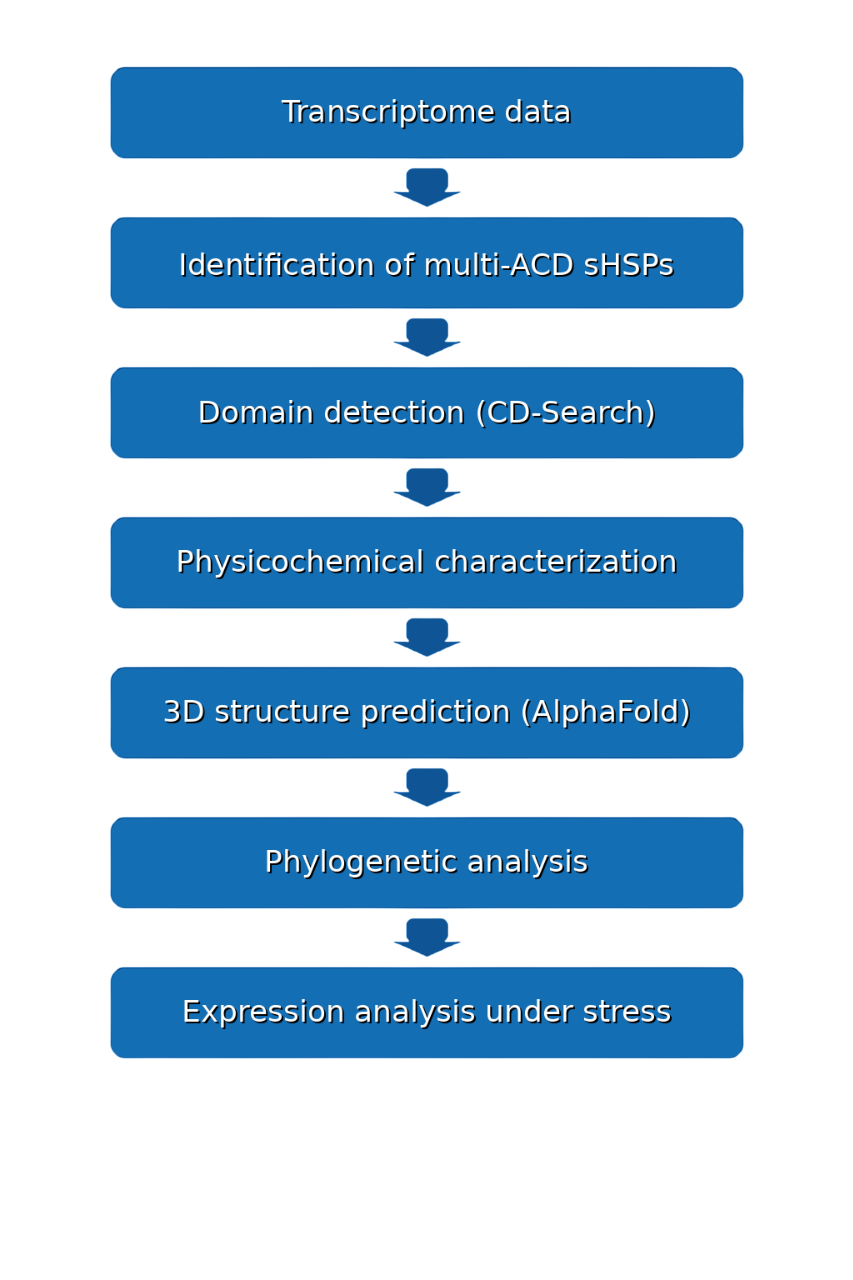


Figure S1. A schematic overview of the analytical workflow used for characterization of multi-ACD sHSPs.


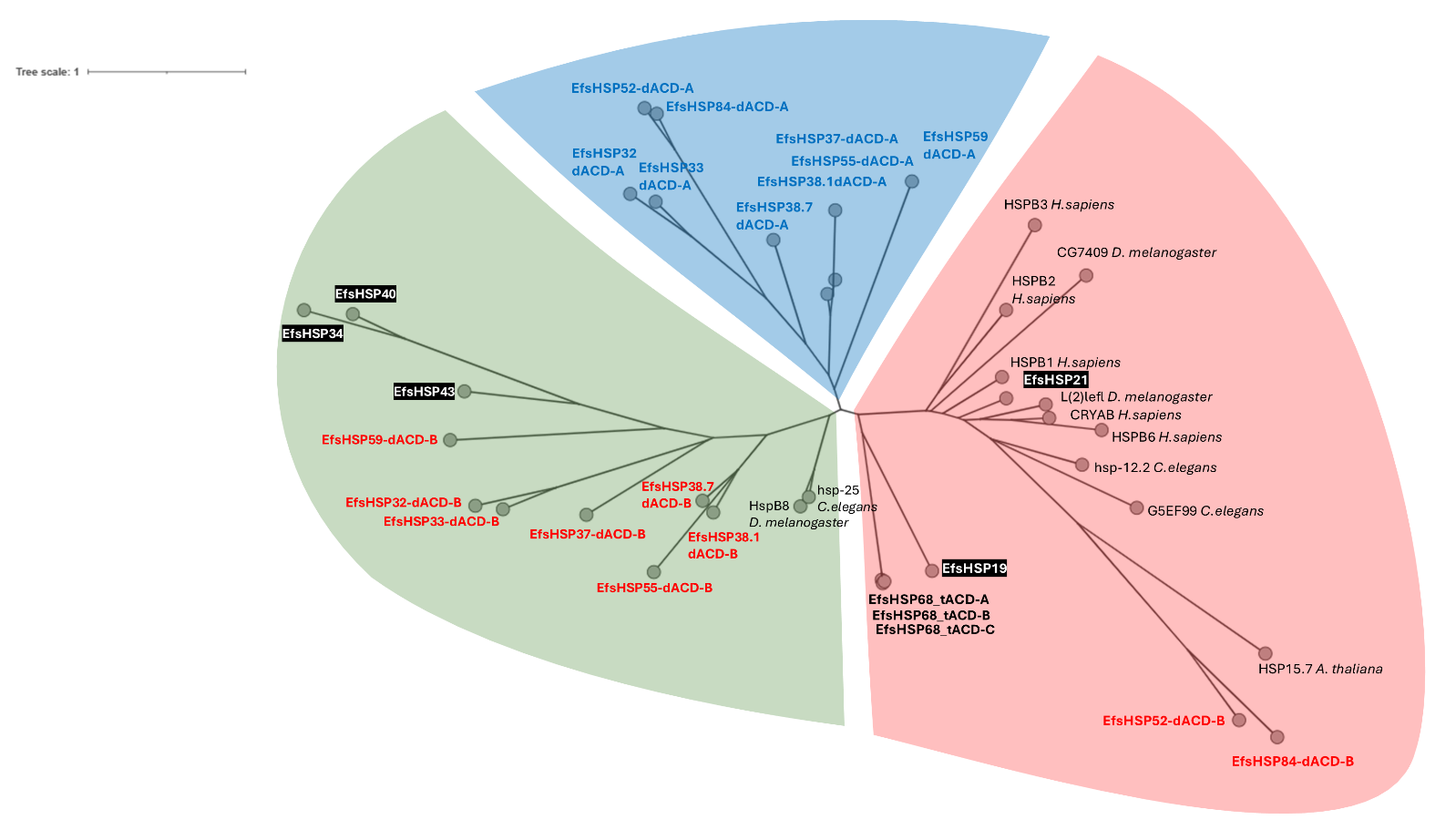


Figure S2. Maximum Likelihood (ML) tree constructed from the multiple sequence alignment of the alpha-crystallin domain (ACD) regions of small heat shock proteins (sHSPs) involved in the study, including sHSPs with dual ACD domain architecture, monomeric sHSPs (black background), triple ACD sHSPs (bold letters), and other well-characterized metazoan sHSPs. In sHSPs with double ACDs, the first ACD regions are represented in blue letters, and the second ACD regions in red letters. Clade colors correspond to those used in De la Fuente and Novo (2022).
